# Genome-Wide Homozygosity Patterns and Evidence for Selection in a Set of European and Near Eastern Horse Breeds

**DOI:** 10.3390/genes10070491

**Published:** 2019-06-28

**Authors:** Gertrud Grilz-Seger, Markus Neuditschko, Anne Ricard, Brandon Velie, Gabriella Lindgren, Matjaz Mesarič, Marko Cotman, Michaela Horna, Max Dobretsberger, Gottfried Brem, Thomas Druml

**Affiliations:** 1 Institute of Animal Breeding and Genetics, University of Veterinary Sciences Vienna, Veterinärplatz 1, 1210 Vienna, Austria; 2 Agroscope, Swiss National Stud Farm, Les Longs Prés, CH-1580 Avenches, Switzerland; 3 UMR 1313 Génétique Animale et Biologie Intégrative, Institut National de la Recherche Agronomique, Domaine de Vilvert, Bat 211, 78352 Jouy-en-Josas, France; 4 Department of Animal Breeding & Genetics, Swedish University of Agricultural Sciences, Ulls väg 26, 750 07 Uppsala, Sweden; 5 School of Life and Environmental Sciences, University of Sydney, Eastern Ave, Sydney, NSW 2006, Australia; 6 Livestock Genetics, Department of Biosystems, KU Leuven, 3001 Leuven, Belgium; 7 Clinic for Reproduction and Large Animals, University of Ljubljana, Veterinary, Faculty, Cesta v Mestni log 47, 1000 Ljubljana, Slovenia; 8 Institute for Preclinical Sciences, University of Ljubljana, Veterinary Faculty, Gerbičeva 60, 1000 Ljubljana, Slovenia; 9 Department of Animal Husbandry, Slovak University of Agriculture in Nitra, Tr. A. Hlinku 2, 949 76 Nitra, Slovakia

**Keywords:** ROH island, selection signature, body size, coat color, autophagy, altitude adaptation, embryonic morphogenesis

## Abstract

Intensive artificial and natural selection have shaped substantial variation among European horse breeds. Whereas most equine selection signature studies employ divergent genetic population structures in order to derive specific inter-breed targets of selection, we screened a total of 1476 horses originating from 12 breeds for the loss of genetic diversity by runs of homozygosity (ROH) utilizing a 670,000 single nucleotide polymorphism (SNP) genotyping array. Overlapping homozygous regions (ROH islands) indicating signatures of selection were identified by breed and similarities/dissimilarities between populations were evaluated. In the entire dataset, 180 ROH islands were identified, whilst 100 islands were breed specific, all other overlapped in 36 genomic regions with at least one ROH island of another breed. Furthermore, two ROH hot spots were determined at horse chromosome 3 (ECA3) and ECA11. Besides the confirmation of previously documented target genes involved in selection for coat color (*MC1R*, *STX17*, *ASIP*), body size (*LCORL/NCAPG*, *ZFAT*, *LASP1*, *HMGA2*), racing ability (*PPARGC1A*), behavioral traits (*GRIN2B*, *NTM/OPCML*) and gait patterns (*DMRT3*), several putative target genes related to embryonic morphogenesis (*HOXB*), energy metabolism (*IGFBP-1*, *IGFBP*-*3*), hair follicle morphogenesis (*KRT25*, *KRT27*, *INTU*) and autophagy (*RALB*) were highlighted. Furthermore, genes were pinpointed which might be involved in environmental adaptation of specific habitats (*UVSSA*, *STXBP4*, *COX11*, *HLF*, *MMD*).

## 1. Introduction

**Figure 1:**
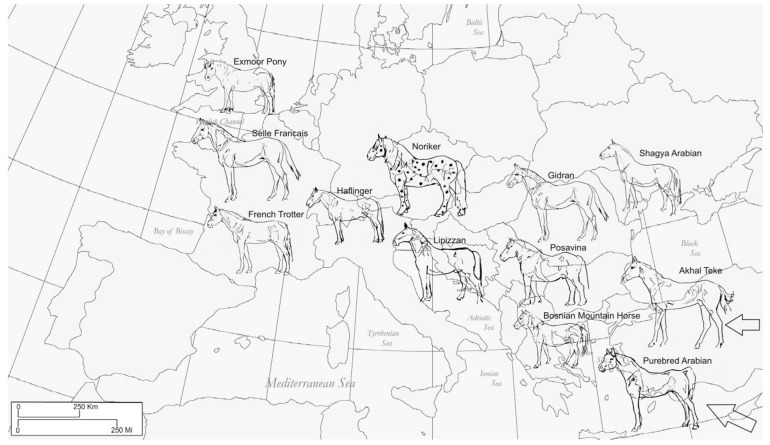
Geographic origin of twelve investigated European and Near Eastern horse breeds (Graphics Thomas Druml).

The scientific interest in the identification of selection signatures in horses successively rose with the enhancement of whole-genome sequencing and analysis methods. A milestone in equine genetics is the population study of Gu et al. [1] that concentrated on the identification of selection targets in thoroughbreds using microsatellite markers. With the availability of cost extensive high throughput single nucleotide polymorphism (SNP) data, several studies were conducted to scan the genome of numerous horse populations for genetic diversity [2,3], and for selection signatures [4,5,6,7]. Furthermore, the capability of archaeogenomics to isolate and analyze ancient DNA enabled the comparison of the ancient equine genome with modern breeds, which highlighted the genomic changes during the domestication process [8,9]. Most of these studies employ methods which ascertain the loss of diversity in order to capture breed divergent patterns of molecular variation, among them F_ST_ statistics being the most popular [10,11]. In other livestock species, authors investigated selection signatures based on common shared runs of homozygosity (ROH islands) [12,13,14,15]. To date, several studies applying this approach on horses were published [16,17,18,19,20,21]. To our knowledge, one of the first equine studies scanning the genome for runs of homozygosity (ROH) in order to detect selection signatures was performed by Metzger et al. [16] using the next generation sequencing data of 10 horses (three horses originating from primitive breeds and seven horses originating from modern breeds). Grilz-Seger et al. [17,18] and Velie et al. [19] investigated ROH island patterns within single breeds using genotype information of the 670k SNP genotyping array. A comparison of overlapping homozygous regions between three different breeds with a small population size using 670k SNP data was performed by Grilz-Seger et al. [20], whilst Nolte et al. [21] investigated four German sport horse breeds based upon 50k SNP data. In this study, we investigated a highly diverse breed panel of 12 European and Near-Eastern horse breeds, comprising in total 1476 horses, for potential signatures of selection utilizing the 670kSNP genotyping array (Table 1). The horses were selected to represent a North–South gradient ranging from Great Britain over Central and South-Eastern Europe to the Near East (Figure 1).

The main objective of this study was to determine levels of autozygosity, with a special focus on overlapping homozygous regions (ROH islands) within and between breeds, in order to identify breed-specific signatures of selection. Furthermore, we conducted a gene ontology (GO) and enrichment analysis and compared and discussed resulting gene ontologies within our breed panel.

## 2. Materials and Methods 

### 2.1. Sampling

The sample comprised 1476 horses from ten European and two Near Eastern horse breeds, whereas the single breeds belonged to different phenotype categories: two heavy draught horse breeds (Noriker, n = 174, Posavina, n = 28), two small sized autochthonous breeds (Exmoor Pony, n = 104, Bosnian Mountain Horse, n = 23), two Arabian breeds (Purebred Arabian, n = 155, Shagya Arabian, n = 32), three Warmblooded breeds (Selle Francais, n = 294, Lipizzan, n = 377, Gidran, n = 20), one racing breed (French Trotter, n = 156), one small to medium sized multipurpose working breed (Haflinger, n = 78) and the Akhal Teke (n = 35) (Table 1). Three of the sampled breeds can be considered as very old (Noriker, Bosnian Mountain Horse and Exmoor Pony) and are kept under semi-feral conditions on a long term. 

The majority of the horses (Haflinger, Noriker, Lipizzan, Posavina, Bosnian Mountain Horse, Gidran and Shagya Arabian) were selected in order to represent the genealogical population structure (sire lines and mare families) of the respective breeds based on pedigree information. For Akhal Teke, French Trotter and Selle Francais, the horses were chosen to be unrelated within two generations. In the Exmoor Pony sample only horses with four generation long pedigrees were included and closely related individuals (full sibs) were excluded. For Lipizzan, Noriker, Posavina and Bosnian Mountain Horse SNP data were available from previous studies [17,18,20]. The samples for Lipizzan, Shagya Arabian, Akhal Teke, Haflinger and Noriker were discussed and approved by the institutional Commission for Ethics and Animal Welfare, University of Veterinary Medicine, Vienna, protocol number: ETK-06/05/2015. The hair samples of Bosnian Mountain, Posavina and Gidran horses were collected during routine procedures for studbook registration by the Institute for Breeding and Health Care of Horses of the Veterinary Faculty, Ljubljana. The samples of Selle Francais, Purebred Arabian and French Trotter horses were collected in accordance with generalized scheme of preferences (GSP) guidelines and national legislation and the Exmoor Pony samples were approved by the Ethics Committee for Animal Experiments in Uppsala, Sweden (Number: C 121/14).

### 2.2. SNP Genotyping

The SNP genotypes of the 1476 horses were derived using the Affymetrix Axiom™ Equine genotyping array [3]. The chromosomal position of the SNPs was determined based on EquCab2 reference genome [31]. We did not consider SNPs positioned on the sex chromosomes (X: 28 017 SNPs and Y: 1 SNP) and SNPs without known chromosomal position (30,864 SNPs). SNPs with more than 10% missing genotypes were excluded. This resulted in a total of 611,914 SNPs that passed quality control and were used for further genetic analyses.

### 2.3. Population Stratification and ROH Analysis

In order to illustrate the population structure, we applied a principal component analysis (PCA) based upon the genetic relationship matrix (G) with pairwise identities by state (IBS) between horses as provided by PLINK v.1.7 [32]. The PCA scatter plot was performed using the R-platform (www.r-project.org).

ROH segments were determined with an overlapping window approach implemented in PLINK v1.7 [32] based on the following settings: minimum SNP density was set to one SNP per 50 kb, with a maximum gap length of 100 kb. The final segments were called runs of homozygosity (ROH) if the minimum length of the homozygous segment was greater than 500 kb and constituted more than 80 homozygous SNPs, whilst one heterozygote and two missing genotypes were permitted within each segment.

The distribution of ROH segments across the genome was visualized using the R-package detectROHs (www.r-project.org). Putative ROH islands were determined based on overlapping homozygous regions, shared by more than 50% of studied horses within each breed [12]. ROH islands which occurred only in one single breed were defined as “breed-specific” or “private”. Resulting ROH islands were checked for overlaps between breeds, whereas no threshold for the minimal overlapping length size was applied. 

Further statistical analyses, graphical representations and data preparation were performed using the software package SAS v.9.1 [33]. In order to provide a general overview, we plotted all chromosomes and illustrated length and position of ROH islands with boxes and pinpointed location of genes as vertical lines with either: (a) known functions, (b) associated with phenotypes in horses, (c) highlighted in selection signature studies, (d) with singular occurrence in ROH islands, and (e) genes, highlighted by GO analysis.

### 2.4. Gene Ontology Analysis

The equine Ensembl database EquCab2 was used to identify genes located in ROH islands, available at www.ensembl.org. For the determination of gene ontology (GO) terms and KEGG (Kyoto Encyclopaedia of Genes and Genomes) pathways of identified annotated genes, the open source database for annotation, visualization and integrated discovery (DAVID) v6.8 package [34] was used. For the GO analysis the equine annotation file as background and a significance threshold of *p* < 0.05, correcting for multiple testing applying Bonferroni-Holm test, were chosen.

## 3. Results

### 3.1. Population Stratification

**Figure 2:**
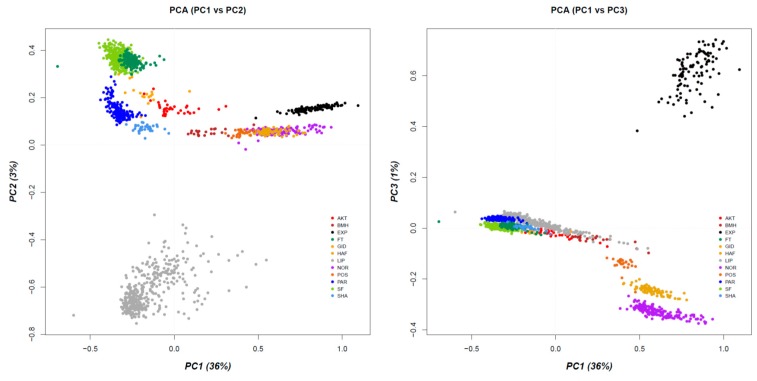
Principal component analysis (PCA) scatterplot illustrating the population stratification of 1,476 horses originating from 12 breeds. On the left visualization of PC1 versus PC2, together explaining 39% of genetic variance, and on the right visualization of PC1 versus PC3 together explaining 37% of variation, are presented. (AKT = Akhal Teke, BMH = Bosnian Mountain Horse, EXP = Exmoor Pony, FT = French Trotter, GID = Gidran, HAF = Haflinger, LIP = Lipizzan, NOR = Noriker, POS = Posavina, PAR = Purebred Arabian, SF = Selle Francais and SHA = Shagya Arabian).

The population structure of the studied horse breeds was ascertained with a PCA scatter plot as shown in Figure 2. Based upon the genetic pairwise identity-by-state (IBS) distance the first principal component 1 (PC1), accounting for 36% of the total variance, divided the 12 breeds into two main groups: (a) draught horse and Pony breeds (Noriker, Haflinger, Posavina and Exmoor Pony) and (b) performing and oriental breeds (Purebred Arabians, Shagya Arabians, Akhal Teke, Gidran, Selle Francais, French Trotter, Lipizzan and Bosnian Mountain Horse). Principal component 2 (PC2), accounting for 3% of the total variance, clearly separated the Lipizzan horses from the other breeds and simultaneously provided a fine-scale population structure of the oriental breeds (Purebred Arabian, Shagya Arabian, Gidran and Akhal Teke) by dividing them from the French Trotter and Selle Francais, whilst the latter two showed a high level of genetic relatedness. Visualization of PC1 versus PC3 (accounting for 1% of the total variance) illustrated the genetic relationship between the breeds and highlighted that the draught horse breeds gradually overlap along PC1 in direction from heavy to lighter type and geographically from North to South (Noriker, Haflinger, Posavina), whilst the Bosnian Mountain Horse built the hub to the oriental breed cluster. Compared to PC2, Exmoor Pony built a distinct cluster on PC3, whilst the Lipizzan horses were allocated next to Purebred Arabian and Shagya Arabian. Furthermore, it can be noticed that the draught horses were clearly divided along small to bigger size (Posavina, Haflinger, Noriker).

### 3.2. ROH Island Pattern and Distribution

Mean genome length covered by runs of homozygosity (S_ROH_) ranged on population level from 192.7 Mb to 506.1 Mb (Table 2). The highest values were assigned to the Exmoor Pony (506.1 Mb), the Arabian group (Purebred Arabian—368.1 Mb, Shagya Arabian—355.1 Mb, Gidran—321.9 Mb) and the French Trotter (352.8 Mb). 

Medium S_ROH_ levels around 290 Mb were found in the Lipizzan, Selle Francais, Haflinger and Bosnian Mountain Horse, followed by the Akhal Teke (246.1 Mb) and Noriker (215.5 Mb). The lowest values were identified in the Posavina (192.7 Mb). The highest number and total length of overlapping homozygous regions were expressed by Exmoor Pony (33 islands, 24.5 Mb) followed by Shagya Arabian (32 islands, 22.4 Mb), Gidran (30 islands, 19.2 Mb) and French Trotter (22 islands, 20.5 Mb) (Table 2). Low numbers of ROH islands were detected in Lipizzan (5 islands, 1.9 Mb), Selle Francais (five islands, 2.1 Mb) and Posavina (five islands, 2.2 Mb) (Table 2, Supplementary File 1). In general, the total ROH island length per breed was consistent with its mean S_ROH_, except for the Lipizzan and Selle Francais, where the length of five overlapping homozygous regions comprised 1.9 Mb, respectively 2.1 Mb in contrast to a medium S_ROH_ of 297 Mb (Table 2).

From a total of 180 identified ROH islands 100 were private for a specific breed (Table 2), whilst all other islands overlapped in 36 genomic regions and were shared by at least two breeds (Table 3). High numbers of private ROH islands were present in Exmoor Pony (28 out of 33), Shagya Arabian (23 out of 32), Gidran (18 out of 30) and in French Trotter (14 out 22) (Table 3). Low numbers of breed specific ROH islands were determined in Lipizzan, Noriker, Posavina and Selle Francais where one out of five homozygous regions was solely found within the respective breed. Within Haflinger no private islands were detected, as all islands overlapped with homozygous regions of other breeds. 

The Exmoor Pony exhibited no common homozygous region with Akhal Teke and the performing breeds. The ROH islands of the Bosnian Mountain Horse did not match with those of the Shagya Arabians and those of the performance breeds, and the French Trotter had no common island with Lipizzan, draught horse breeds, Exmoor Pony and Bosnian Mountain Horse. A summary for all ROH islands per breed is presented in Supplementary File 1 and Supplementary File 2.

The ROH islands were unequally distributed across the genome. Nine chromosomes were ROH island cold spots. Among all breeds no ROH islands were located on the following chromosomes: ECA10, ECA12, ECA13, ECA20, ECA21, ECA24, ECA26, ECA29 and ECA31. 

**Figure 3:**
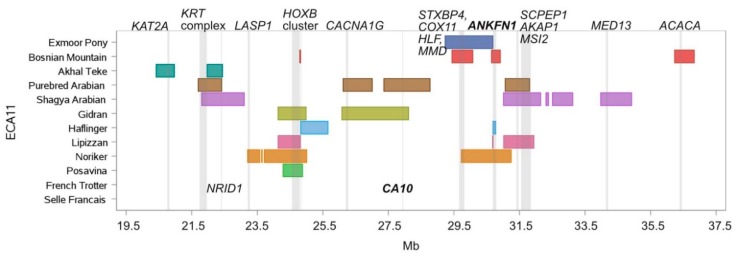
ROH islands on ECA11 between 19.5 Mb and 37.5 Mb. Location and length of ROH islands (shared by more than 50% of individuals per breed) per breed are illustrated as horizontal boxes, location of genes of specific interest are indicated as vertical lines.

ECA11 can be characterized as a ROH island hot spot (Figure 3), as all breeds except French Trotter and Selle Francais had at least one up to five ROH islands located in this region (ECA11: 20–37 Mb). Six islands were breed-specific in: Akhal Teke, Noriker, Shagya Arabian and Bosnian Mountain Horse. The overlapping homozygous regions (Table 3) can be assigned into four different groups: a) the orientalized group, including Akhal Teke, Purebred Arabian, Shagya Arabian from 21.8 Mb to 22.4 Mb, and Purebred Arabian and Gidran from 26.1 Mb to 28.1 Mb, b) breeds originating from the geographical region of the former Austro-Hungarian empire at position 24.2–24.8 Mb, c) autochthonous breeds (Bosnian Mountain Horse, Exmoor Pony and Noriker) with an overlapping homozygous region between 29.7 Mb and 30.1 Mb, d) mixed group including Noriker, Bosnian Mountain Horse, Exmoor Pony, Haflinger, Shagya Arabian, Purebred Arabian and Lipizzan horses with overlapping ROH islands from 30.6 Mb to 31.8 Mb (Figure 3).

The ROH islands of the Arabian breeds overlapped in a genomic region where several members of the *KRT* complex are located (Table 2). The gene *LASP1* (LIM and SH3 protein 1), associated with body size [35], was found in a 235 kb long private ROH island of the Noriker, which harbored nine additional genes. A gene group with fundamental impact on embryonic morphogenesis is the homeobox B (*HOXB*) cluster [36]. This gene cluster was present in up to 1.2 Mb long ROH islands on ECA11 in Noriker, Posavina, Lipizzan and Gidran (Figure 3). The autochthonous breeds (Exmoor Pony, Bosnian Mountain Horse and Noriker) shared a common homozygous region harboring the genes *STXBP4* (syntaxin-binding protein 4), *COX11* (cytochrome c oxidase copper chaperone COX11), *HLF* (HLF transcription factor), and *MMD* (monocyte to macrophage differentiation associated). The islands of the Exmoor Pony and the Noriker exceeded in length up to the gene *ANKFN1* (ankyrin repeat and fibronectin type III domain containing 1), which was also present in the ROH islands of Lipizzan, Bosnian Mountain Horses and Haflinger (Figure 3). The Arabian breeds and the Lipizzan overlapped in a region containing among six annotated genes, the genes *SCPEP1* (serine carboxypeptidase 1), *AKAP1* (A-kinase anchoring protein 1) and *MSI2* (musashi RNA binding protein 2).

**Figure 4:**
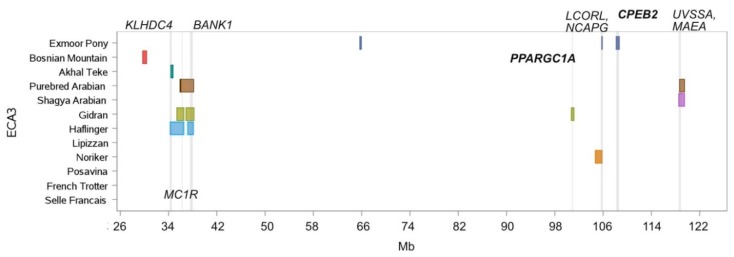
ROH islands on ECA3 between 26 Mb and 122 Mb. Location and length of ROH islands (shared by more than 50% of individuals per breed) per breed are illustrated as horizontal boxes, location of genes of specific interest are indicated as vertical lines.

A second ROH island hot spot was identified on ECA3 in several genomic regions around the genes *KLHDC4* (Kelch domain containing 4), *MC1R* (melanocortin 1 receptor), *BANK1* (B cell scaffold protein with ankyrin repeats 1) and *UVSSA* (UV stimulated scaffold protein A) for breeds derived or influenced by the oriental gene pool (Purebred Arabian, Shagya Arabian, Haflinger and Akhal Teke) (Figure 4). The longest island covered the gene *MC1R* and reached up to 2.2 Mb length in the Haflinger sample (Figure 4). The Arabian breeds exhibited an ROH island at the *UVSSA* locus. Around the *LCORL/NCAPG* locus (ligand dependent nuclear receptor corepressor like/non-SMC condensin I complex subunit G), which was associated with body size [35], another homozygous region was shared by the Exmoor Pony and the Noriker horse. Within the Gidran sample, a private island containing *PPARGC1A* (peroxisome proliferator-activated receptor gamma, coactivator 1 alpha), a gene undergone positive selection in Thoroughbreds [1], was detected. 

**Figure 5:**
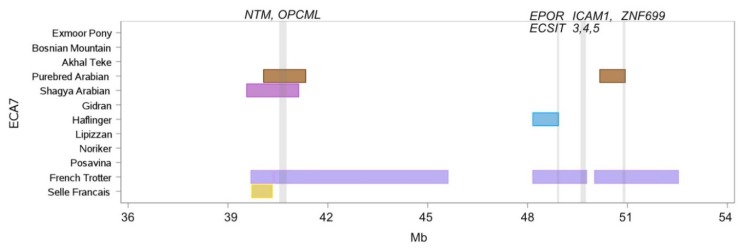
ROH islands on ECA7 between 36 Mb and 54 Mb. Location and length of ROH islands (shared by more than 50% of individuals per breed) per breed are illustrated as horizontal boxes, location of genes of specific interest are indicated as vertical lines.

ROH islands on ECA4 (Supplementary File 2) and ECA7 (Figure 5) were ROH island hot spots predominantly for the oriental group (Purebred Arabian, Shagya Arabian, Gidran and Akhal Teke) and the two performing breeds Selle Francais and French Trotter. The longest homozygous region was found in the French Trotter on ECA7, where four ROH islands were located between 39.6 Mb and 52.5 Mb, comprising together a length of 10.1 Mb which is comparable to 50% of the total length of ROH islands in this breed (Figure 5). Within the same region the Purebred Arabian, Shagya Arabian, Selle Francais and Haflinger overlapped with up to two (Purebred Arabian) shorter ROH islands.

The Selle Francais had, although characterized by a medium genome-wide S_ROH_ of 297 Mb, only five ROH islands in total. The island on ECA7 completely overlapped with the French Trotter specific island at the identical position but not in frequency, as only a small fragment exceeded the 50% threshold (Supplementary File 3). A homozygous region at position 39.5–41.1 Mb was also present in the Purebred and Shagya Arabian and harbored the genes *NTM* (neurotrimin) and *OPCML* (opioid-binding protein/cell adhesion molecule). Out of 22 ROH islands 14 were private within the French Trotter. Among them was an island on ECA23, which harbored the gene *DMRT3* (doublesex and mab-3 related transcription factor 3) (Supplementary File 2). A mutation in *DMRT3* is responsible for the ability to perform alternate gaits [37]. This gene/island was only detected for the French Trotter within the entire sample.

The autochthonous breeds (Exmoor Pony, Noriker, Posavina and Bosnian Mountain Horse) shared three overlapping ROH islands located on three different chromosomes. Besides the aforementioned island on ECA3 containing the gene *NCAPG* (shared by Noriker and Exmoor Pony) (Figure 4), and the island on ECA11 harboring the genes *STXBP4*, *COX11*, *HLF*, and *MMD* (shared by Noriker, Exmoor Pony and Bosnian Mountain Horse) (Figure 3), another common island on ECA9 was identified (Supplementary File 2). This island at position 75.0–75.4 Mb contains the gene *ZFAT* (zinc finger and AT-hook domain) and it was present in the Noriker and Posavina sample (Table 3). 

**Figure 6:**
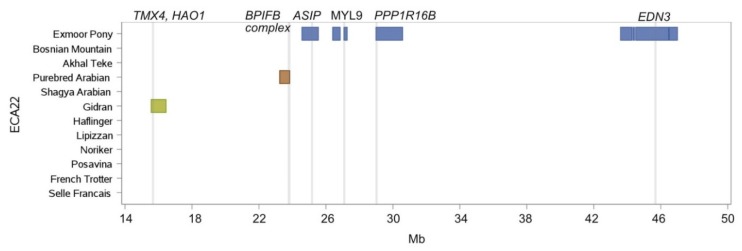
ROH islands on ECA22 between 14 Mb and 50 Mb. Location and length of ROH islands (shared by more than 50% of individuals per breed) per breed are illustrated as horizontal boxes, location of genes of specific interest are indicated as vertical lines.

The high genetic distance of the Exmoor Pony to the other breeds was also evident in the ROH island pattern, where 28 from 33 islands were solely detected within this breed (Table 2). ECA22 was an ROH island hot spot for this British Pony breed covering eight islands between 24.5 Mb and 46.9 Mb, which comprised together a length of 4.7 Mb (Figure 6). The island between 24.5 Mb and 25.5 Mb harbored among 14 other annotated genes, the gene *ASIP* (agouti-signaling-protein) responsible for bay coat color [38].

**Figure 7:**
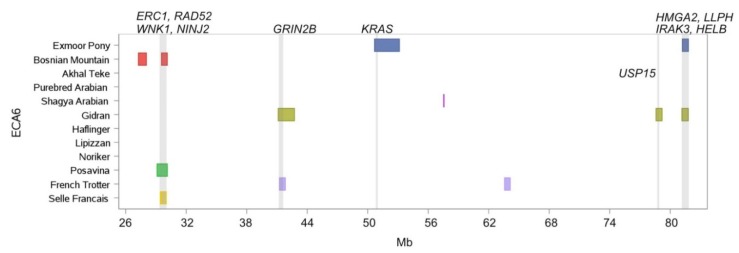
ROH islands on ECA6 between 26 Mb and 80 Mb. Location and length of ROH islands (shared by more than 50% of individuals per breed) per breed are illustrated as horizontal boxes, location of genes of specific interest are indicated as vertical lines.

Beside the aforementioned chromosomes ECA3, ECA4, ECA7, ECA9, and ECA11, ROH islands overlapped in genomic regions on seven further chromosomes (ECA1, ECA6, ECA15, ECA18, ECA19, ECA23, ECA25). Akhal Teke and Bosnian Mountain Horse shared one homozygous region on ECA1 at position 148.4 Mb - 148.1 Mb enclosing one single gene (*EIF2AK4*, eukaryotic translation initiation factor 2 alpha kinase 4). On ECA6 the Posavina, the Bosnian Mountain Horse and the Selle Francais shared an island containing the genes *ERC1* (ELKS/RAB6-interacting/CAST family member 1), *RAD52* (RAD52 homolog), *WNK1* (WNK lysine deficient protein kinase 1) and *NINJ2* (ninjurin 2) (Figure 7). The gene *GRIN2B* (glutamate ionotropic receptor NMDA type subunit 2B) was located in an island of the French Trotter and the Gidran. On the same chromosome the Exmoor Pony exhibited together with the Gidran a ROH island, harboring the genes *HMGA2* (high mobility group AT-hook 2), *LLPH* (LLP homolog, long-term synaptic facilitation factor), *IRAK3* (interleukin 1 receptor associated kinase 3) and *HELB* (DNA helicase B) (Figure 7).

**Figure 8:**
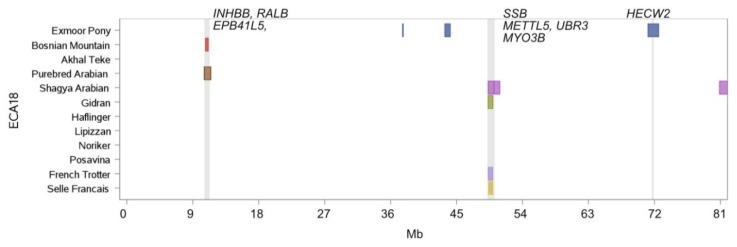
ROH islands on ECA18 between 1 Mb and 81 Mb. Location and length of ROH islands (shared by more than 50% of individuals per breed) per breed are illustrated as horizontal boxes, location of genes of specific interest are indicated as vertical lines.

On ECA18 the French Trotter, Selle Francais, Gidran and Shagya Arabian shared a ROH island at position 49.3 Mb - 49.4 Mb containing the genes *SSB* (small RNA binding exonuclease protection factor La), *METTL5* (methyltransferase like 5), *UBR3* (ubiquitin protein ligase E3 component n-recognin 3), and *MYO3B* (myosin IIIB). Furthermore, the genes *RALB* (Ras-like G-protein) and *INHBB* (Inhibin subunit beta B) were identified in a shared homozygous region of the Bosnian Mountain Horse and Purebred Arabian (Figure 8).

### 3.3. Genotype Frequencies of Genes Involved in Coat Color and Body Size

In six breeds of the dataset, coat color represents an explicit breeding objective. In the Gidran and Haflinger, chestnut coat color is fixed. The desired color for the Exmoor Pony is brown combined with mealy areas around the eyes, muzzle, elbow and flanks. Lipizzan horses have been selected for their gray color for 150 years and the frequency of gray phenotypes reaches up to 98% [18] and the breeding program of the Noriker horse is based upon the selection for six different coat colors (bay, black, chestnut, leopard, roan and tobiano). 

We identified a ROH island containing *MC1R*, responsible for chestnut coat color, in the Gidran (1.1 Mb length), Haflinger (2.2 Mb length) and the Purebred Arabian (2.1 Mb length) samples. Genotype data for *MC1R* was available and is summarized for each breed in Supplementary File 4. All breeds with a high frequency of chestnut coat color had an additional ROH island on ECA3 (Figure 4), which was scalariform structured. The core ROH island harbored the genes *NFKB1* (Nuclear factor NF-kappa-B p105 subunit), *SLC39A8* (solute carrier family 39 member 8) and *BANK1* and was embedded in an up to 2.1 Mb long ROH island in Purebred Arabians. Highest frequencies (95%) for this core island were observed in the Gidran. In the Gidran sample (100% homozygous *T/T* on *MC1R*) an additional ROH island on ECA25 was identified, harboring the *STX17* gene responsible for gray coat color. This homozygous region was 403.5 kb long and contained the genes *STX17*, *NR4A3*, *ERP44* and *INVS*. Purebred and Shagya Arabian had a ROH island on ECA3 containing the genes *MAEA* (macrophage erythroblast attacher) () and *UVSSA* (Figure 4), which plays an important role in the nucleotide excision repair (NER) pathway responsible for the reparation of DNA damage caused by UV radiation [39]. From a previous study [18] we know, that the Lipizzan had an ROH island around *UVSSA*, which is too small to be detected with a 500 kb window. We extracted the four available SNPs within the *UVSSA* gene for the entire sample and revealed for two non-synonymous SNPs (AX-103191894 in intron 7 and AX-104669126 in intron 6 ) distinct genotype distributions between the breeds, corresponding with the geographical dispersion area of origin (Supplementary File 5). 

Within the Exmoor Pony we further identified an ROH island on ECA22 containing among 14 annotated genes, the gene *ASIP* (agouti signaling protein) and an island on ECA1 harboring the genes *OCA2* and *HERC2* (Supplementary File 2). *OCA2/HERC2* were associated with eye pigmentation (blue, green, hazel eyes), lighter skin pigmentation, blond and red hair in humans [40]. 

The genome-wide ROH island scan revealed several loci (*LCORL/NCAPG*, *ZFAT*, *LASP1* and *HMGA2*) associated with body size [35] and height at withers, back and croup length (*ZFAT*) [41]. Body size associated loci were embedded in islands of the draught horse breeds Noriker (*LCORL/NCAPG*, *ZFAT* and *LASP1*), Posavina (*ZFAT*) and in the Exmoor Pony (*LCORL/NCAPG* and *HMGA2*). Interestingly also in Gidran a 639 kb long ROH island containing *HMGA2* together with the genes *LLPH* and *IRAK3* was identified. Three out of the four SNPs associated with body size according to Makvandi-Nejad et al. [35] are available on the applied genotyping array. From a previous study [17] we know that the majority of Noriker horses are homozygous for the “Big”-alleles of the SNPs near *LCORL/NCAPG*, *ZFAT* and *LASP1* and that the Posavina horse shifts to the “small” allele of the *LCORL/NCAPG* gene. We extracted the three size associated SNPs for the Exmoor Pony and the Bosnian Mountain Horse, a breed that is known to be selected for a height at withers around 135 cm on a long term. The majority of Bosnian Mountain Horses (57–87%) were homozygous for the “small” alleles in all three loci (Supplementary File 6). The Exmoor Pony was monomorphic for the “small” allele near the *LCORL/NCAPG* gene and 92% of horses were also homozygous for the “small” allele near *ZFAT*. Deviation from Hardy-Weinberg equilibrium was observed for the *ZFAT* locus in Exmoor Pony (*p* > 0.014) and revealed on-going selection towards small body size. 

### 3.4. Gene Ontology and Enrichment Analysis

From the 91 breed specific GO terms and six KEGG pathways determined, 14 GO terms remained significant after correction for multiple testing in Noriker, Lipizzan, Posavina, Gidran, Shagya Arabian, Purebred Arabian, and French Trotter. A detailed description of enrichment analysis for each breed is given in Supplementary File 7. Several GO terms were shared by more than one breed (Table 4). High significance levels (Bonferroni adjusted *p*-value<0.05) were reached for the GO terms anterior/posterior pattern specification (GO:0009952), embryonic skeletal system morphogenesis (GO:0048704) and sequence-specific DNA binding (GO:0043565), mainly based upon the *HOXB*-cluster in the breeds Gidran, Lipizzan, Posavina and Noriker. For Purebred and Shagya Arabian, several members of the *KRT*-complex retained the term intermediate filament (GO:0005882) (Table 4). 

Two breed specific annotation clusters were significantly enriched after correcting for multiple testing (*p* < 0.05) within the French Trotter (GO:0005178, integrin binding, based upon *ICAM1*, *ICAM4*, *ICAM5* and *ICAM3*) and the Purebred Arabian (GO:0005198, structural molecule activity, based upon *KRT*-complex) (Supplementary File 7).

## 4. Discussion

Due to its huge economic impact and its global genetic introgression into a wide range of contemporary horse breeds, the English Thoroughbred has been in the focus of genomic publications for the last decades [1,42,43,44,45]. For a range of Middle European horse breeds, the Arabian horse represented an equivalent source of genetic improvement, which in a historical context preceded the refinement by the Thoroughbred. For nine of the analysed breeds in this study (Purebred Arabian, Shagya Arabian, Akhal Teke, Lipizzan, Bosnian Mountain Horse, Posavina, Haflinger, Noriker, and Exmoor Pony) the absence of introgression of the English Thoroughbred is historically documented. Hence, we only identified few ROH islands in genomic regions that were targets of positive selection in the English Thoroughbred [1]. 

ECA3 and ECA11 were common ROH island hotspots in nearly all breeds of our dataset, except for French Trotter and Selle Francais, harboring genes involved in coat color (ECA3: *MC1R*), size (ECA3: *LCORL/NCAPG*, ECA11: *LASP1*), NER pathway (ECA3: *UVSSA*), embryonic skeletal morphogenesis (ECA11: *HOXB*-cluster) and coat texture (ECA11: *KRT*-complex). Recently, Gurgul et al. [7] identified ECA11 as a major selection signature hotspot in six Polish horse breeds with a similar type composition (two draught horse breeds, two autochthonous/primitive breeds, one oriental and one Warmblood horse breed). The authors focused on diversifying selection signatures by grouping the breeds into different major horse type categories (light, primitive and draught type). Based upon this grouping they detected a strong signal between draught and primitive breeds on ECA11 within the *LASP1* locus associated with body size [35], a region which overlapped with a ROH island (23.2–23.4 Mb) detected in the Noriker sample. 

Nolte et al. [21] identified selection signatures on ECA11 for the *HOXB*-cluster in four German sport horse breeds (Hannoveraner, Holsteiner, Oldenburger and Trakehner). The *HOXB*-cluster was present in ROH islands of Lipizzan, Noriker, Posavina and Gidran horses, and was significantly highlighted by the GO terms embryonic skeletal morphogenesis and anterior/posterior pattern specification in all four breeds. *HOX* genes play a fundamental role for morphological diversity in animals and for the control of axial morphology along the anterior-posterior body axis [36]. On the same chromosome an up to 1.3 Mb long ROH island was detected in the Arabian breeds and the Achal Teke (slightly under the threshold) containing several members of the *KRT* complex (*KRT* 12, 20, 23-28), which were also pinpointed by GO analysis revealing the terms hair follicle morphogenesis and intermediate filament. Keratin proteins represent a major part of the protective matrix of the skin, hair and horn in mammals [46], and *KRT25* and *SP6* were associated with curly coat in Bashkir Curly Horses and Missouri Foxtrotters by Thomer et al. [47]. A variant (KRT25:p.R89H) in the *KRT25* gene was found to be responsible for the curly phenotype in North-American and French horses [48]. The keratin driven GO term intermediate filament was also documented in the aforementioned study by Nolte et al. [21]. 

Coat color, one of the major documented targets of selection during the domestication process [49,50], represents a central breeding objective in five of the investigated breeds (Lipizzan, Haflinger, Gidran, Exmoor Pony and Noriker). We found an up to 2.5 Mb long homozygous region on ECA3 in Gidran, Haflinger and Purebred Arabian horses around the *MC1R* locus responsible for chestnut coat color [51]. A haplotype around *MC1R* was previously reported by McCue et al. [4] and Petersen et al. [5]. The investigated breeds with high frequencies of chestnut phenotype harbored two additional islands on ECA3, in which among others the genes *NFKB1*, *SLC39A8*, *BANK1*, *UVSSA* and *MAEA* are located. In two linked non-synonymous intronic SNPs of the *UVSSA* gene oriental breeds exhibited convergent minor allelic and genotype frequencies. Interestingly, the Lipizzan where the majority of the gene pool originates from Spanish Horses [52] clustered to the Oriental group. A mutation in the *UVSSA* gene has been found to be causative for the autosomal recessive disorder UV-sensitive syndrome in humans and together with *USP7* it mediates the transcription-coupled nucleotide excision repair [39]. The *SLC39A8* gene encodes a transmembrane protein that acts as a transporter of several cations, including zinc. Zinc is essential for various cellular functions and zinc deficiency causes a broad range of disorders in humans and animals, such as growth retardation, immune dysfunctions, diarrhea, and skin diseases [53]. A mutation in *BANK1* was associated with the autoimmune disorder systemic lupus erythematous in humans [54]. Finally, the gene *NFKB1*, a member of the NF-ĸB transcription factor family, regulates a large number of genes involved in inflammation, cell cycle and cell survival. The NF-ĸB signaling pathway is important for the maintenance of immune homeostasis in epithelial tissues, especially in the regulation of homeostasis and inflammation in skin [55]. Our study revealed very high frequencies (up to 95%) of chestnut horses harboring *NFKB1*, *SLC39A8*, *BANK1* and *UVSSA* in ROH islands, and indicated evidence that these genes may be involved in the reported higher susceptibility of chestnut horses for skin disorders [56]. Additionally, we identified within the Gidran sample an ROH island containing the *STX17* haplotype, responsible for gray coat color [57]. This island was nearly identical in length with the ROH island of Lipizzan horses, containing the genes *NR4A3*, *STX17*, *ERP44* and *INVS* [18]. The gray haplotype in non-gray horses was firstly reported by Pielberg et al. [57,58] and predominantly found in Oriental horses. All these findings support a connection between the gray locus and the Oriental horse gene pool. 

In Exmoor Ponies we identified a ROH island on ECA22 containing the *ASIP* gene responsible for bay coat color [38] and one island on ECA1 harboring the genes *OCA2* and *HERC2*. In humans several SNPs in the *OCA2* and *HERC2* genes were associated with eye, skin, and hair pigmentation [40]. Fernández et al. [59] found evidence that *OCA2* has an effect on skin color intensity in red strain of Iberian pigs. *OCA2* and *HERC2* were suggested as candidate genes for the leopard spotting pattern [60] and for the equine “Tiger-eye” phenotype by Kowalski and Bellone [61] but could not be confirmed [62]. To our knowledge, no investigation on the influence of *OCA2/HERC2* on the mealy phenotype, as exhibited by the Exmoor Pony, has been conducted. Mealy coat color in the Exmoor breed was recently proposed to be linked to the *EDN3* locus in a study focusing on harness trotting traits [63]. As a homozygous region around *EDN3* was also found in non-mealy Hungarian Lipizzan subpopulation [18], we suggest further research including phenotypic data. We did not identify ROH islands around the *KITLG* gene on ECA28 which was previously reported to undergo selection in several horse breeds [8,16]. From the various functions of *KITLG*, also known as mast cell growth factor, which is involved in processes affecting melanogenesis, haematopoiesis and gametogenesis, melanogenesis has been in the focus of scientific research in equine genetics [16,64,65,66].

As already mentioned, most of our samples were not influenced by the English Thoroughbred gene pool. Nevertheless, the genes *STXBP4* and *COX11*, which were undergoing positive selection in Thoroughbreds [1] and were also listed among other 123 genes exposed to selection during domestication process [8], were present in ROH islands of the Bosnian Mountain Horse, Exmoor Pony and Noriker. Other Thoroughbred related genomic regions were identified in the Anglo-Arabian Gidran breed. The gene *PPARGC1A*, a candidate gene for physical performance in Thoroughbreds [1] was located in a 444 kb long ROH island of the Gidran. *PPARGC1A* encodes PGC-1α, which is a transcriptional co-activator that regulates genes involved in energy metabolism and mitochondrial biogenesis through expression of nuclear signaling proteins [67]. The overrepresentation of genes involved in transcription was underlined in four GO terms (transcription factor complex, nucleus, sequence-specific DNA binding, DNA-binding transcription factor activity). Additionally, seven ROH island on ECA4 were identified within the Gidran sample, three islands directly overlapped with one of the genes *CDK6*, *HDAC9*, and *FOXP2*. Pilegaard et al. [68] reported that endurance exercise induces transient transcriptional activation of the PGC-1α in human skeletal muscle, and Eivers et al. [69] postulated a PGC-1α exercise induced role of *HDAC9* in the myogenesis of horses. 

In general, ECA4 and ECA7 were ROH island hotspots in the performing and oriental breeds, which shared homozygous segments in six genomic regions and partially overlapped with ROH islands found in Hannoveraner, Holsteiner, Oldenburger and Trakehner [21]. The authors pinpointed selection signatures for *IGFBP-1*, *3* and *4*. *IGFBP-1* and *IGFBP-3*, which were located in ROH islands of the French Trotter, were highlighted by the GO terms IGF I binding (GO:00312994) and IGF II binding (GO:0031995). 

For the French Trotter, which genealogically has been influenced by the Standardbred and therefore simultaneously by the Thoroughbred founder gene pool, a closer link to Thoroughbred specific selection signatures was not observed. Due to the specification on differing locomotion patterns (trot and pace), selection favored different genomic regions on a long term. We identified in the French Trotter a ROH island on ECA23 containing the *DMRT3* gene. A mutation in *DMRT3* is causative for ambling locomotion pattern and favorable effect on harness racing performance in horses [37]. Single SNPs in *DMRT3* were also associated with age-dependent trotting ability by Ricard et al. [70]. 

ECA7 was a ROH island hotspot within the French Trotter, which exhibited four ROH islands between 39.6 Mb and 52.5 Mb, comprising together a length of 10.1 Mb. Petersen et al. [5] reported extended haplotypes for Standardbred on ECA7. The homozygous region of the French Trotter overlapped at position 39.5–41.1 Mb with islands of Shagya and Purebred Arabians, in which among others the genes *NTM* and *OPCML* are located. These genes, associated with intelligence and cognitive functions in humans [71,72], and hypothesized to modulate temperament in horses were highlighted by Gurgul et al. [7] focusing on signals of diversifying selection between light versus draught horses. Additionally, the authors found high linkage disequilibrium (LD) in the chromosomal area between 40.1 Mb and 52.2 Mb in Arabian and Malopolski horse, which corresponded with the extended ROH island of the French Trotter at 39.6 Mb and 52.5 Mb [7]. Avila et al. [73] also proposed that *NTM* might influence temperament of pleasure horses. We identified a further gene on ECA6, which might be involved in behavioral traits. The gene *GRINB2*, located in the ROH islands of French Trotter and Gidran, is involved in enhanced learning ability in mice [74] and it was associated with career earnings in the Swedish Coldblooded Trotter, and further proposed to affect learnability in horses [75]. 

Body size and height of withers are important breeding objectives and are well investigated. Makvandi-Nejad et al. [35] associated four loci (*LCORL/NCAPG*, *ZFAT*, *LASP1* and *HGMA2*) with body size in horses. Signer-Hasler et al. [41] further identified SNPs in the *ZFAT* gene, which were associated with height at withers. Selection signatures around *NCAPG* on ECA3 and *LASP1* on ECA11 were identified by Petersen et al. [5] in pony and draught horse breeds and by Gurgul et al. [7] in primitive (Huzul and Konik) and draught horse breeds. Our results were in concordance with these findings and we identified ROH islands for size associated loci in draught horse breeds (Noriker (*LCORL/NCAPG*, *ZFAT* and *LASP1*) and Posavina (*ZFAT*)) and Exmoor Pony (*LCORL/NCAPG* and *HMGA2*). Interestingly also the Gidran, a multipurpose riding horse, exhibited a 636 kb long island on ECA 6 containing *HMGA2*. 

Three breeds in our study (Noriker, Bosnian Mountain Horse and Exmoor Pony) can be considered as very old autochthonous breeds, adapted to semi-feral rearing conditions on a long term. The Noriker and the Bosnian Mountain Horse originate from the Alpine/Dinaric region, an environment characterized by high altitude, difficult terrain, cold temperature and deprivation of nutrition, especially in winter. High altitude exposes animals to permanent oxidative stress and results in adaption of the blood, cardiovascular, pulmonary and muscle systems. In all three breeds, we identified a common ROH island on ECA11 containing the genes *STXBP4*, *COX11*, *HLF* and *MMD* (additional *TMEM100* and *PCTP* in Exmoor Ponies). All these genes were highlighted in a study investigating adaptation to high altitude in the Andean Horse [76]. Environmental conditions and different performance disciplines can affect similar physiological mechanisms. Three further genes (*METTL5*, *UBR3* and *MYO3B*) highlighted in the high-altitude study of Hendrickson [76] were also present in ROH islands on ECA18 for the breeds French Trotter, Gidran, Selle Francais and Shagya Arabian. Autophagy, a strategy to cope with starvation, is in the focus of human genetic research [77,78]. Within the Bosnian Mountain Horse and the Purebred Arabians, we identified an ROH island containing the genes *RALB* and *INHBB*. Bodemann et al. [79] described *RALB* (Ras-like G-protein) as a regulatory switch to promote autophagosome biogenesis. We found three ROH islands in Purebred and Shagya Arabians pinpointing the genes *LAPTM4B* and *IDUA*. *LAPTM4B* (Lysosomal protein transmembrane 4 beta) promotes autophagosome and lysosome fusion [80]. Finally, the gene *CALCOCO2*, involved in selective autophagy [81], was present in ROH islands of Posavina, Gidran, Bosnian Mountain Horse, Haflinger and Noriker, whereas for the latter additionally the genes *OSBPL*, *PIP4K2B* were highlighted in the GO term autophagosome. The accumulation of genes involved in autophagy in breeds, which are known to be selected for low-input and extensive rearing systems, needs further investigations including phenotypic data. 

Within the Exmoor Pony, no autophagy related genes were highlighted. A well-known evolutionary adaption in mammalians, generally known as island or Foster’s rule, is the decrease of body size due to limited space and nutrition [82]. Among the investigated horse breeds the Exmoor Pony exhibits the smallest body size at an average height at withers of 120 cm. This characteristic was also pinpointed by the genotype results for size associated loci. Additionally, the 662 kb long ROH island on ECA6 in Exmoor Pony at position 81.12-81.80 Mb harboring the genes *HMGA2*, *LLPH*, *IRAK3* and *HELB* supports these considerations. Norton et al. [83] identified ancestral haplotypes between ECA6:81.16-81.58 associated with height and baseline insulin values in Welsh ponies and supposed *HMGA2* and *IRAK3* as candidate genes involved in the equine metabolic syndrome.

Summarizing the results of our study, we found genes located within ROH islands and shared by more than 50% of a breed, for coat color in Purebred Arabian, Haflinger, Gidran (*MC1R*), in Lipizzan and Gidran (*STX17*) and in Exmoor Pony (*ASIP*) and additionally for coat quality (*KRT*-complex) in Purebred Arabian and Shagya Arabian. Size associated loci were pinpointed in draught horses (Noriker (*LCORL/NCAPG*, *ZFAT* and *LASP1*), Posavina (*ZFAT*)), Exmoor Pony (*LCORL/NCAPG* and *HMGA2*) and Gidran (*HMGA2*). Within Gidran and French Trotter selection for behavioral traits was indicated by *GRINB2* and *NTM/OPCML*. Selection targets for racing performance, exercise and gait pattern was shown for French Trotter (*DMRT3*, *IGFBP-1* and *IGFBP-3*) and for Gidran (*PPARGC1A*), whereas conformation breeding was pinpointed by the *HOXB*-cluster in four breeds (Lipizzan, Noriker, Posavina and Gidran). Several genes related to organism response to oxidative stress were embedded in ROH islands for the autochthonous breeds Bosnian Mountain Horse, Noriker and Exmoor Pony (*COX11*, *STXBP4*, *HLF* and *MMD*) and in the oriental and performing breeds (*SSB*, *METTL5*, *UBR3* and *MYO3B*). Finally, genes involved in autophagy were underlined for Purebred Arabian, Gidran, Bosnian Mountain Horse and Noriker (*CALCOCO2*, *RALB* and *LAPTM4B*).

## 5. Conclusions

This study highlighted several genes which were located in regions putatively undergoing artificial and/or natural selection. Many of these genes were causative or associated with traits which are part of breeding objectives in the respective breeds. Besides artificial selection, the post-domestication process has not prevented natural influences. Our study revealed several genes involved in adaption to high altitude and genes which may play a role in the adaption to a lack of nutrition in horses. To validate the presented putative areas of selection, we suggest further investigation including phenotype information. According to the methodical point of view, we demonstrated that ROH island analysis offers the possibility to identify common targets of selection in divergent breeds. Therefore, this approach provides a wider perspective and an enhanced insight into the complexity of biological processes and physiological functions of equines.

## Figures and Tables

**Table 1:** Characterization of breeds, population history, sample location and number of sampled horses (N).

Breed/Classification	Gene Pool	Region of Origin/Sample Origin	Population Notes and Census
Exmoor Pony small sized, multipurpose working horse	Native English breed	British Isles/UK	Closed stud book since 1921, ca. 2.000 animals [2]
Selle Francais Riding horse	French Trotter, Thoroughbred, Arabian	France/FRA	Stud book founded 1958; ca. 12.700 breeding animals [22]
French Trotter harness racing (trot)	Anglo Norman, Standardbred	France/FRA	Stud book closed since 1937, allows Standardbred; 15.500 breeding animals [23]
Lipizzan riding, driving	Old-Spanish and Oriental breeds	Austro—Hungarian/Lipizzan Stud farms AT, SLK, HUN, HR	Founded 1580, closed studbook since 1880; ca. 2.000 breeding animals in European state stud farms [24]
Noriker heavy working draught horse	Native Austrian breed	Austria/AT	Very old breed, closed stud book since 1880; ca. 4.000 breeding animals [25]
Haflinger light draught horse, multipurpose	Derived from local and Galizian mares, and influenced by Arabian, Gidran, Noriker	Austria/AT	Founded 1898, closed studbook since 1928; ca. 6.000 breeding animals in Austria [26]
Posavina small draught horse, heavy working	Native Croatian breed, influenced by Ardenner, Bosnian Mountain Horse	Croatia/SLO	Closed studbook since 1994; ca. 600 breeding animals in Slovenia [27]
Gidran multipurpose riding horse	Arabian, English Thoroughbred, Old-Spanish	Stud farms Mezöhegyes, Radautz (Austro - Hungarian empire)/HUN	Closed studbook since 1860, introgression of some Arabian and Thoroughbred horses; ca. 250 breeding animals in Hungary [28]
Shagya Arabian endurance and multipurpose riding horse	Original Arabians from Syria, influence by Russian founder mares	Stud farms Babolna, Mezöhegyes, Radautz (Austro - Hungarian empire)/SLK	Closed studbook since 1830, Purebred Arabians allowed; ca. 2.000 breeding animals [29]
Bosnian Mountain Horse small multipurpose working horse	Native breed of the Balkan Peninsula, influenced by Arabian	Bosnia and Herzegovina/BIH, SLO	Purebred, very old breed; 114 registered animals in the International Association of Bosnian Mountain Horse Breeders [30]
Purebred Arabian endurance and multipurpose riding horse	Original Arabian	Egypt, Near East/FRA	Purebred, very old breed; worldwide
Akhal Teke Endurance horse	Native Middle East	Turkmenistan/RUS	Purebred, stud book closed 1941; ca. 3.500 breeding animals [2]

**Table 2:** Mean genome length covered by runs of homozygosity S_ROH_ values (in Mb), total length of overlapping homozygous regions (ROH islands) (in Mb), number of ROH islands and number of annotated genes within ROH islands per breed and number of private ROH islands per breed.

Breed	S_ROH_ Mb	Sum ROH Islands Mb	n ROH Islands	n Genes in ROH Islands	n Private ROH Islands
Exmoor Pony	506.1	24.5	33	171	28 on ECA1,2,3,4,6,16,18,19,22,23,28,30
French Trotter	352.8	20.5	22	156	14 on ECA2,4,6,7,8,9,15,23
Selle Francais	297.1	2.1	5	8	1 on ECA1
Lipizzan	297.0	1.9	5	23	1 on ECA14
Noriker	215.5	5.0	5	50	1 on ECA11
Posavina	192.7	2.2	5	16	1 on ECA28
Haflinger	279.7	4.9	5	60	-
Gidran	321.9	19.2	30	143	18 on ECA2,3,4,6,9,14,17,22,25,27,28
Purebred Arabian	368.1	15.5	19	141	5 on ECA2,4,5,14,22
Shagya Arabian	355.1	22.4	32	242	23 on ECA1,2,6,8,9,11,15,16,18,19, 23,25
Akhal Teke	246.1	4.8	9	90	4 on ECA1,11,19
Bosnian Mountain Horse	296.3	4.2	10	35	4 on ECA3,6,11,23

**Table 3:** Overlaps of runs of homozygosity (ROH) islands (begin and end in Mb) between breeds and annotated genes.

Chr.	Begin	End	Breed Shared ROH Islands	Annotated Genes within ROH Islands
1	148,443.474	148,516.796	Akhal Teke, Bosnian Mountain Horse	*EIF2AK4*
3	34,386.385	34,732.351	Akhal Teke, Haflinger	*KLHDC4, SLC7A5, CA5A, BANP*
3	36,047.262	36,548.306	Gidran, Haflinger, Purebred Arabian	*CDK10, SPATA2L, ZNF276, VPS9D1, FANCA, SPIRE2, TCF25, DBNDD1, GAS8, MC1R, DEF8*
3	37,179.496	38,127.255	Gidran, Haflinger, Purebred Arabian	*MANBA, NFKB1, SLC39A8, BANK1*
3	105,767.304	105,832.553	Exmoor Pony, Noriker	*NCAPG, DCAF16*
3	118,669.793	118,769.903	Lipizzan, Shagya Arabian, Purebred Arabian	*UVSSA, MAEA*
3	118,809.979	118,893.880	Lipizzan, Shagya Arabian, Purebred Arabian	*CTBP1*
3	118,659.800	119,479.623	Shagya Arabian, Purebred Arabian	*UVSSA, MAEA, CTBP1, SPON2, FGFRL1, IDUA, SLC26A1, DGKQ, TMEM175, GAK, CPLX1, PCGF3, SLC49A3, MYL5, ATP5ME, PDE6B, PIGG*
4	15,163.634	15,817.917	Akhal Teke, French Trotter	*NUDCD3, NPC1L1, DDX56, TMED4, OGDH, ZMIZ2, H2AFV, MYO1G, CCM2, TBRG4, RAMO3*
4	50,883.038	50,904.590	Gidran, Shagya Arabian	*HDAC9*
4	52,692.165	52,840.002	Gidran, French Trotter	*ABCB5*
6	29,424.361	30,040.425	Bosnian Mountain Horse, Posavina, Selle Francais	*ERC1, RAD52, WNK1, NINJ2*
6	41,240.508	41,843.164	Gidran, French Trotter	*GRIN2B*
6	81,156.975	81,795.964	Exmoor Pony, Gidran	*HMGA2, LLPH, IRAK3*
7	39,587.420	41,127.737	French Trotter, Purebred Arabian, Shagya Arabian	*NTM, OPCML*
7	48,157.328	48,930.905	French Trotter, Haflinger	*ACP5, ELOF1, CNN1, ECSIT, ZNF653, PRKCSH, RGL3, EPOR, SWSAP1*
7	50,167.155	50,932.454	French Trotter, Purebred Arabian	*ZNF699*
9	31,329.939	32,034.084	Purebred Arabian, Shagya Arabian	*PCMTD1, PXDNL*
9	44,411.007	44,562.960	Purebred Arabian, Shagya Arabian, Exmoor Pony, (Haflinger)	*KCNS2*
9	75,054.137	75,434.658	Noriker, Posavina	*ZFAT*
11	21,801.064	21,938.218	Purebred Arabian, Shagya Arabian	*KRT28, KRT27, KRT26, KRT25, KRT24*
11	21,966.387	22,416.682	Akhal-Teke, Purebred Arabian, Shagya Arabian	*SMARCE1, CCR7, TNS4, GFBP4, TOP2A, RARA, CDC6, WIPF2, RAPGEFL1, CASC3, MSL1, NR1D1, THRA*
11	24,285.730	24,816.254	Noriker, Posavina, Gidran, Lipizzan	*HOXB1, HOXB2, HOXB3, HOXB5, HOXB6, HOXB7, HOXB8, HOXB13, TTLL6*
11	24,793.573	24,822.752	Noriker, Posavina, Bosnian Mountain Horse, Haflinger, Gidran	*CALCOCO2*
11	26,117.473	27,008.802	Gidran, Purebred Arabian	*ACSF2, CHAD, RSAD1, MYCABPAP, EPN3, SPATA20, CACNA1G, ABCC3, ANKRD40, LUC7L3, ANKRD40CL, WFIKKN2, TOB1, SPAG9, NME1-NME2, MBTD1, UTP18*
11	27,363.048	28,118.531	Gidran, Purebred Arabian	*CA10*
11	29,747.581	30,078.455	Bosnian Mountain Horse, Exmoor Pony, Noriker,	*STXBP4, COX11, HLF, MMD*
11	30,690.860	30,774.494	Bosnian Mountain Horse, Haflinger, Lipizzan, Noriker	*ANKFN1*
11	31,062.702	31,250.934	Lipizzan, Noriker, Purebred Arabian, Shagya Arabian	*C11H17orf67*
11	31,062.702	31,810.316	Purebred Arabian, Shagya Arabian, Lipizzan	*C11H17orf67, DGKE, COIL, SCPEP1, AKAP1, MSI2*
15	79,489.871	79,964.006	French Trotter, Shagya Arabian	-
18	10,733.279	11,116.742	Bosnian Mountain Horse, Purebred Arabian	*INHBB, RALB, EPB41L5*
18	49,323.427	49,940.945	French Trotter, Gidran, Selle Francais, Shagya Arabian	*SSB, METTL5, UBR3, MYO3B*
19	50,554.541	50,890.075	Gidran, Purebred Arabian	*CBLB, ALCAM*
23	54,975.635	54,984.719	French Trotter, Gidran	*FGD3*
25	6,409.342	6,812.785	Lipizzan, Gidran	*NR4A3, STX17, ERP44, INVS*

**Table 4:** Gene Ontology terms and Kyoto Encyclopaedia of Genes and Genomes (KEGG) pathways, which were shared by two or more breeds (BH *p*-value = Bonferroni adjusted *p*-value).

**GO Biological process**	**Breeds sharing term**	***p*-value**	**Genes**	**BH *p*-value**
GO:0009952~anterior/posterior pattern specification	Lipizzan/Posavina/Gidran	0.000	*HOXB3, HOXB1, HOXB2, HOXB7, HOXB8, HOXB5, HOXB6*	< = 0.001
	Noriker	0.000	*HOXB3, HOXB1, PCGF2, HOXB2, HOXB7, HOXB8, HOXB5, HOXB6*	
GO:0021570~rhombomere 4 development	Lipizzan/Posavina/Noriker/Gidran	0.003	*HOXB1, HOXB2*	0.06–0.99
GO:0021612~facial nerve structural organization	Lipizzan/Posavina/Noriker/Gidran	0.011	*HOXB1, HOXB2*	0.18–0.98
GO:0048704~embryonic skeletal system morphogenesis	Lipizzan/Posavina/Gidran	0.000	*HOXB3, HOXB1, HOXB2, HOXB7, HOXB8, HOXB5, HOXB6*	<0.001
	Noriker	0.000	*HOXB3, HOXB1, PCGF2, HOXB2, HOXB7, HOXB8, HOXB5, HOXB6*	
GO:0071222~cellular response to lipopolysaccharide	Akhal Teke	0.034	*IL6, NR1D1, RARA*	0.99
	Purebred Arabian	0.011	*NR1D1, NFKB1, RARA, SPON2*	
**GO Cellular component**	**Breeds sharing term**	***p*-value**	**Genes**	**BH *p*-value**
GO:0005654~nucleoplasm	Akhal Teke	0.010	*COASY, CDC6, HSD17B1, BBX, ACLY, BANP, CNP, STAT3, SMARCE1, ZMIZ2, DNAJC7, ATP6V0A1, TOP2A*	0.36–0.57
	Noriker	0.010	*CWC25, MRPL10, HOXB7, PSMB3, SNF8, PNPO, HOXB13, KPNB1, PIP4K2B*	
GO:0005882~intermediate filament	Shagya Arabian	0.002	*KRT26, KRT25, KRT28, KRT27, KRT24*	<0.001–0.02
	Purebred Arabian	0.000	*KRT26, KRT25, KRT28, KRT27, KRT12, KRT20, KRT23, KRT24*	
**GO Molecular function**	**Breeds sharing term**	***p*-value**	**Genes**	**BH *p*-value**
GO:0003700~transcription factor activity, sequence-specific DNA binding	Gidran	0.033	*HOXB2, HOXB7, HOXB8, HOXB6, NFKB1, CBFA2T3, TCF25, FOXP2*	0.11–0.99
	Lipizzan/Posavina	0.007	*HOXB2, HOXB7, HOXB8, HOXB6*	
	Noriker	0.012	*HOXB2, HOXB7, HOXB8, HOXB6, ZFAT*	
GO:0005198~structural molecule activity	Shagya Arabian	0.032	*KRT26, KRT25, KRT28, KRT27, EPB41, KRT24*	0.02–0.99
	Purebred Arabian	0.000	*KRT26, KRT25, KRT28, KRT27, KRT12, KRT20, KRT23, KRT24*	
GO:0015299~solute:proton antiporter activity	Purebred Arabian/Gidran	0.044	*SLC9B1, SLC9B2*	0.99
GO:0043565~sequence-specific DNA binding	Gidran	0.019	*HOXB1, HOXB2, HOXB7, HOXB6, HOXB13, PPARGC1A, FOXP2*	0.007–0.92
	Lipizzan/Posavina/Noriker	0.000	*HOXB1, HOXB2, HOXB7, HOXB6, HOXB13*	

## Data Availability

The primary data of this study are owned by different research groups. Primary data of the breeds Lipizzan, Noriker, Haflinger, Akhal Teke, Shagya Arabian, Gidran, Bosnian Mountain Horse and Posavina are available from project consortium FFG project number 843464, Veterinary University Vienna, Xenogenetik, five European state stud farms and the Austrian and Slovenian Horse breeders Association, but restrictions apply to the availability of these data, which were used under license for the current study, and so are not publicly available. Data are however available from the authors upon reasonable request and with permission of project consortium, FFG project number 843464, Veterinary University Vienna, Xenogenetik and partners. Genotype data for the Exmoor Pony breed will be provided by contacting authors Lindgren/Velie or for a larger data set via the following reference: Velie, B.D.; Shrestha, M.; Franҫois, L.; Schurink, A.; Tesfayonas, Y.G.; Stinckens, A.; Blott, S.; Ducro, B.J.; Mikko, S.; Thomas, R.; Swinburne, J.E.; Sundqvist, M.; Eriksson, S.; Buys, N.; Lindgren, G. Using an inbred horse breed in a high density genome-wide scan for genetic risk factors of insect bite hypersensitivity (IBH). *PLoS One*. **2016**, *11*, e0152966.

## Supplementary Materials

The following are available online at https://www.mdpi.com/2073-4425/10/7/491/s1; Suppl. File 1 S1: ROH islands per breed, Suppl. File 2 S2: Illustrations of chromosomes and ROH islands per each breed, Suppl. File 3 S3: ROH distribution on ECA7 in the breeds French Trotter and Selle Francais, Suppl. File 4 S4: Genotype distribution for the MC1R locus in the twelve studied breeds, Suppl. File 5 S5: Genotype and minor/major allele frequencies of the SNP AX-103191894 in intron 7 of the UVSSA gene, Suppl. File 6 S6: Genotype frequencies of SNPs at the loci LCORL/NCAPG, ZFAT and LASP1 for the breeds Bosnian Mountain Horse and Exmoor Pony, Suppl. File 7 S7: Gene Ontology and enrichment analysis.
