# Supplementary material for: Genome-Wide Homozygosity Patterns and Evidence for Selection in a Set of European and Near Eastern Horse Breeds
Source: Genes (Basel). 2019 Jun 28;10(7):491. doi: 10.3390/genes10070491 (PMC6679042; doi:10.3390/genes10070491)
Supplement: Supplementary file 1 [file genes-10-00491-s001.zip › Supplementary_file2.docx]

**Supplementary File 2.** Illustrations of chromosomes and ROH islands per breed. Annotated genes within ROH islands are pinpointed which meet following criteria, either: a) functions are known, b) associated with phenotypes in horses, c) highlighted in selection signature studies, d) genes with singular occurrence within a ROH island (bold letters), e) highlighted by GO analysis.


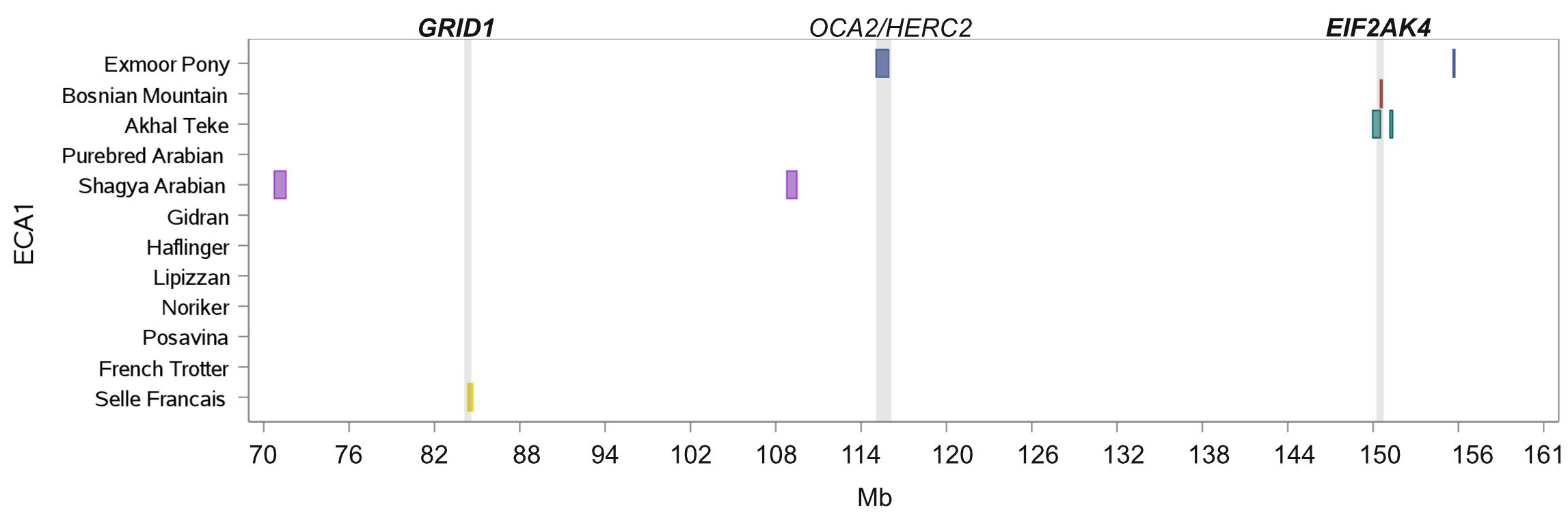


*ECA1: Plot of ROH islands (shared by more than 50% of individuals per breed) per breed including genes of specific interest.*


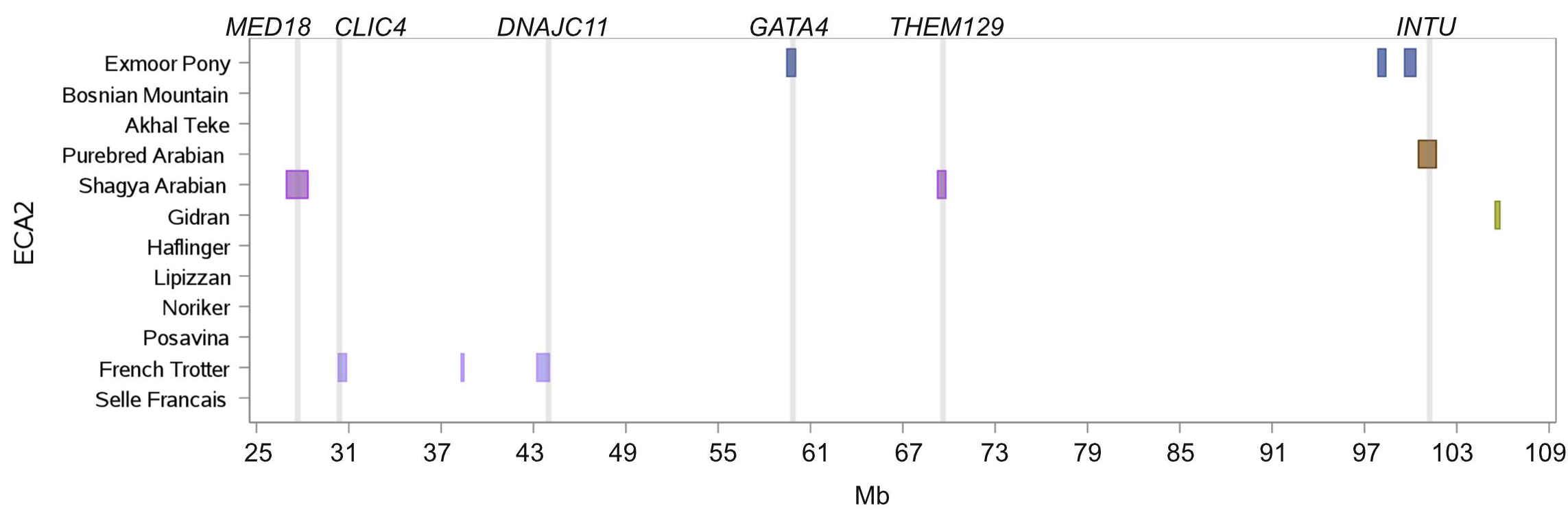


*ECA2: Plot of ROH islands (shared by more than 50% of individuals per breed) per breed including genes of specific interest.*


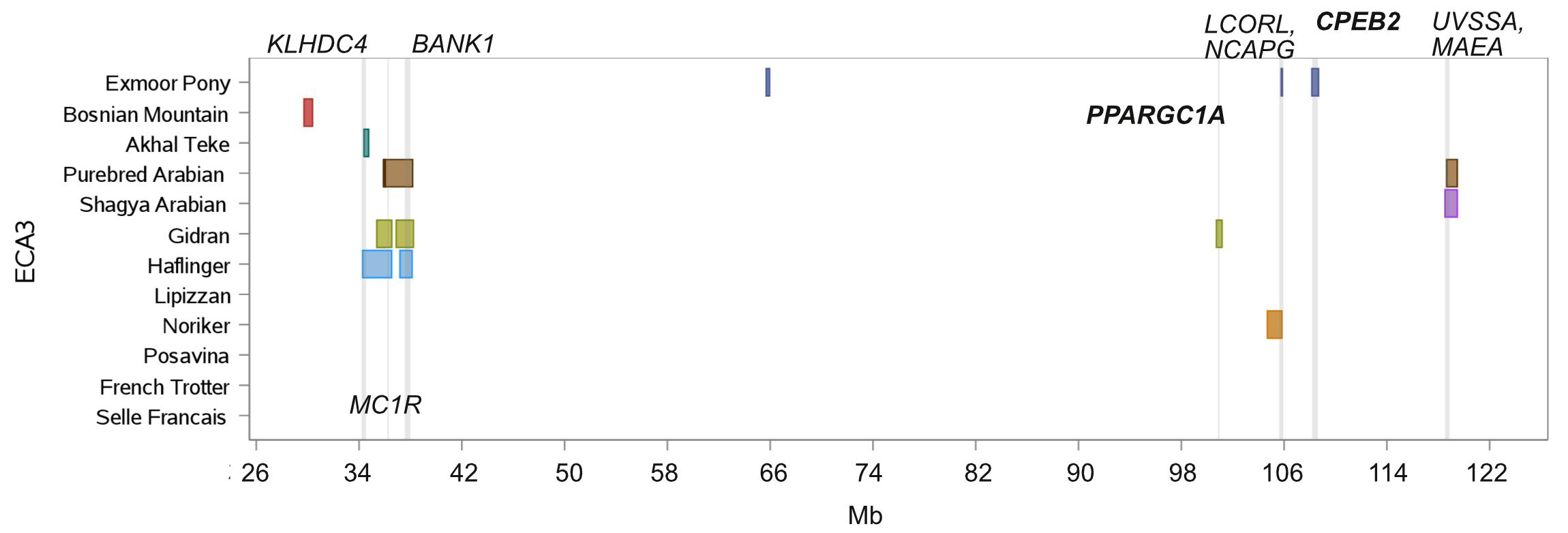


*ECA3: Plot of ROH islands (shared by more than 50% of individuals per breed) per breed including genes of specific interest.*


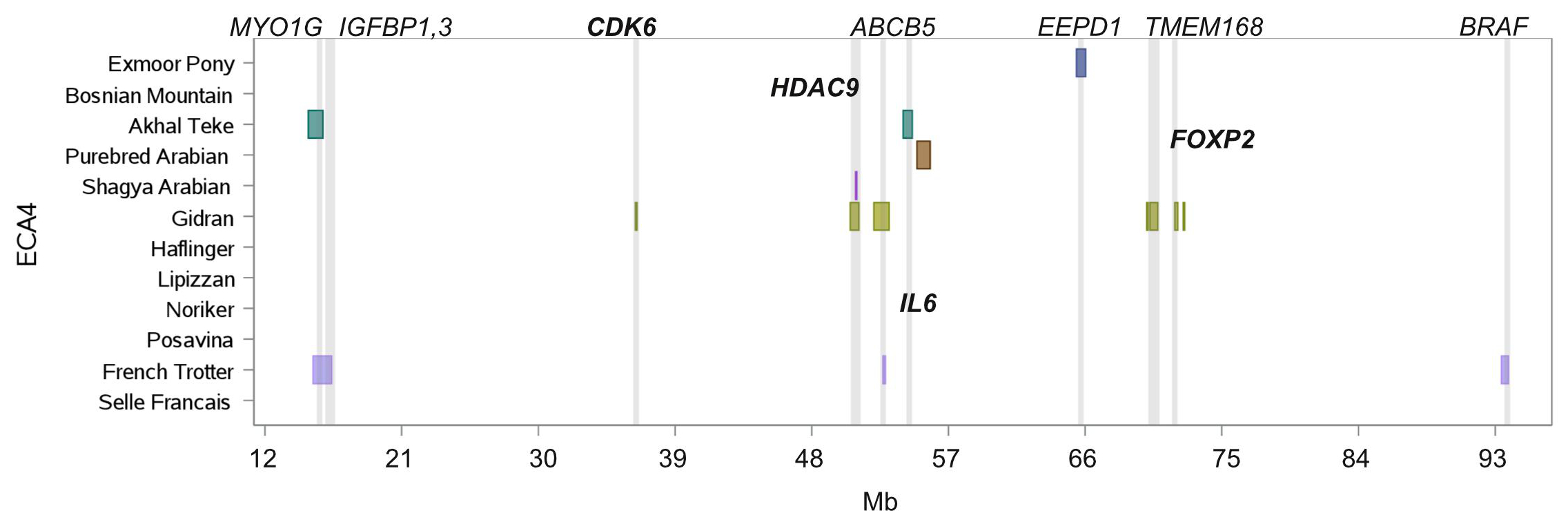


*ECA4: Plot of ROH islands (shared by more than 50% of individuals per breed) per breed including genes of specific interest.*


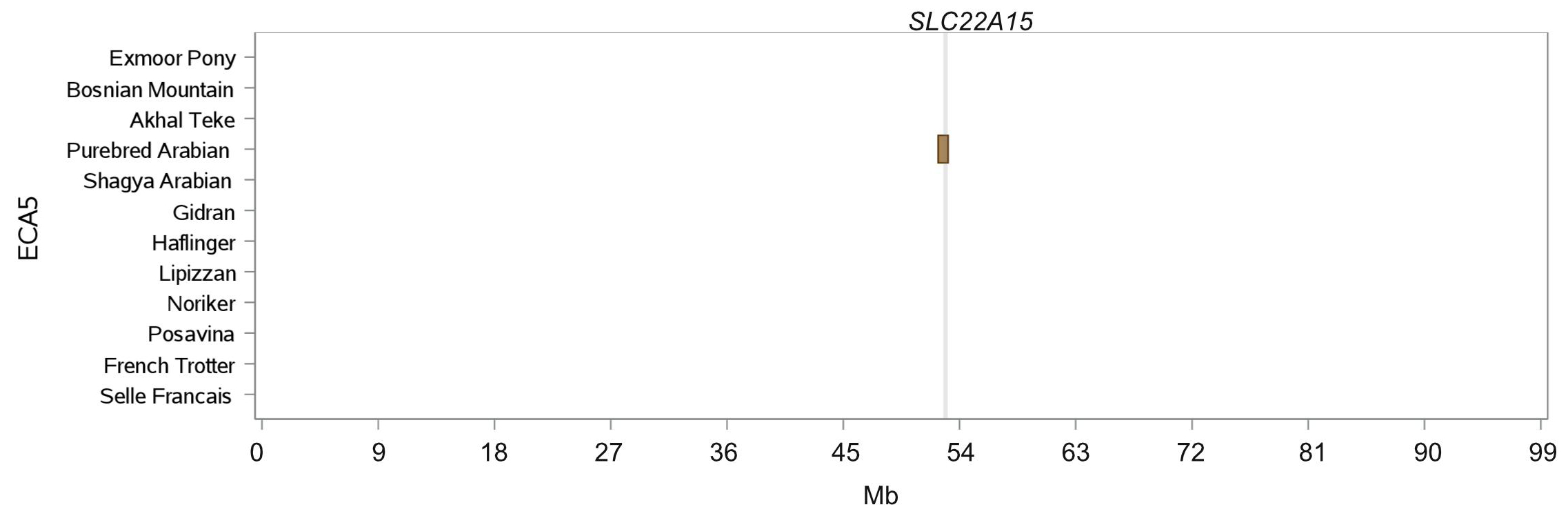


*ECA5: Plot of ROH islands (shared by more than 50% of individuals per breed) per breed including genes of specific interest.*


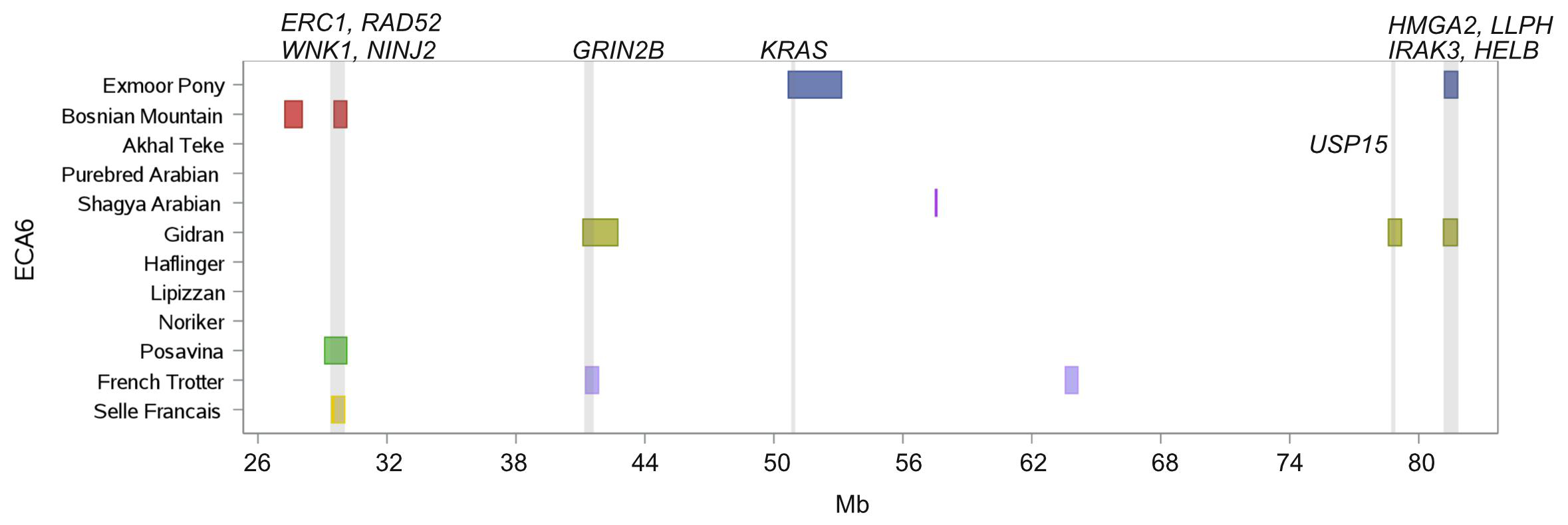


*ECA6: Plot of ROH islands (shared by more than 50% of individuals per breed) per breed including genes of specific interest.*


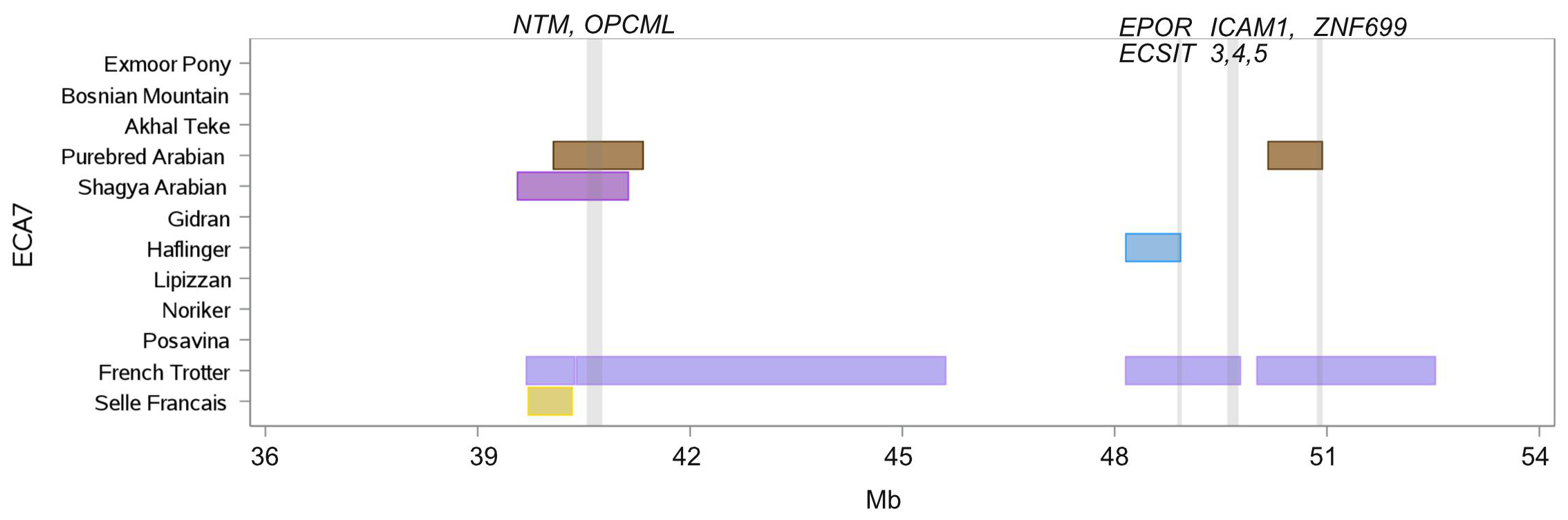


*ECA7: Plot of ROH islands (shared by more than 50% of individuals per breed) per breed including genes of specific interest.*


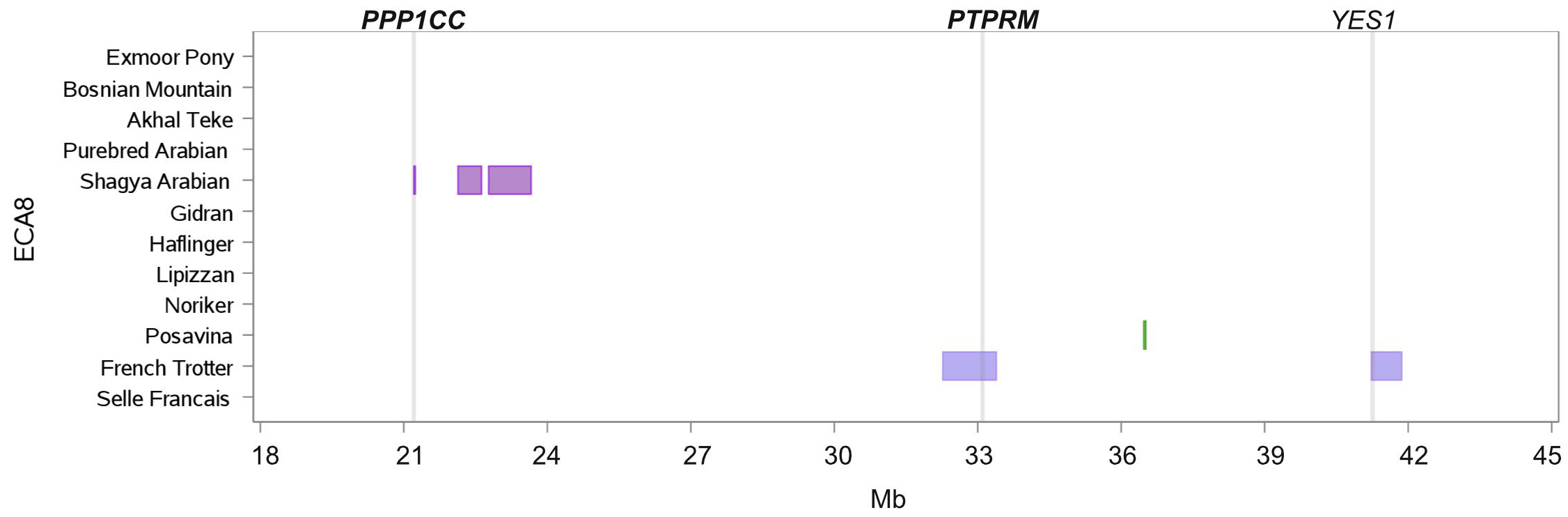


*ECA8: Plot of ROH islands (shared by more than 50% of individuals per breed) per breed including genes of specific interest.*


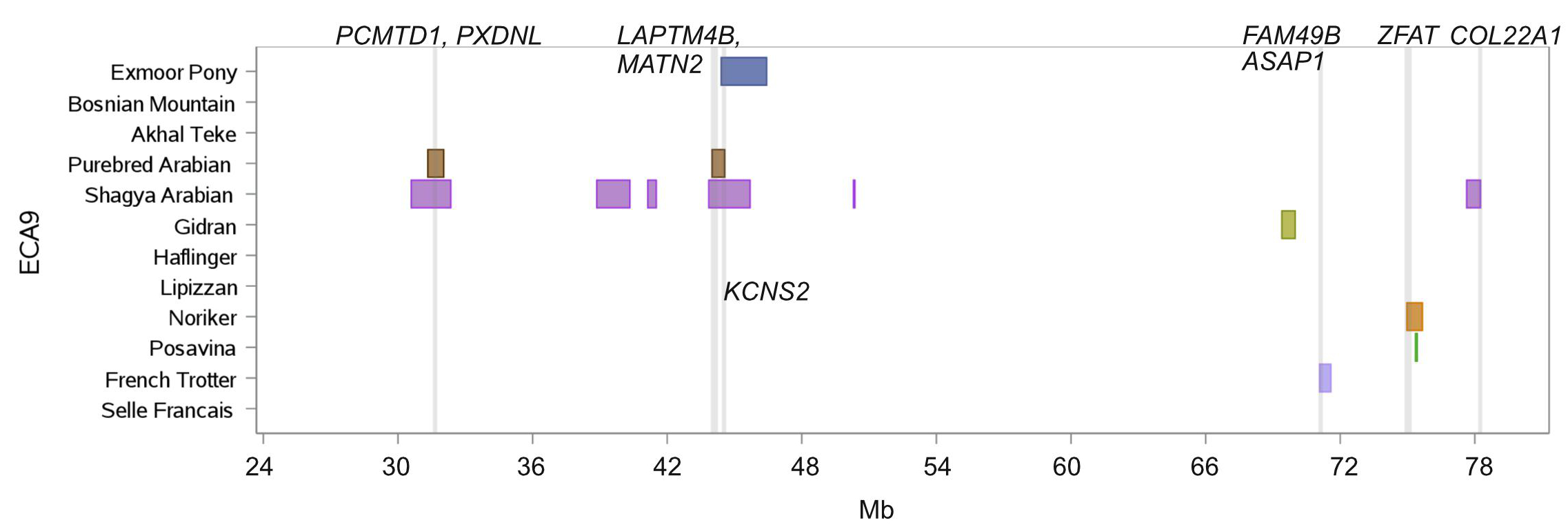


*ECA9: Plot of ROH islands (shared by more than 50% of individuals per breed) per breed including genes of specific interest.*


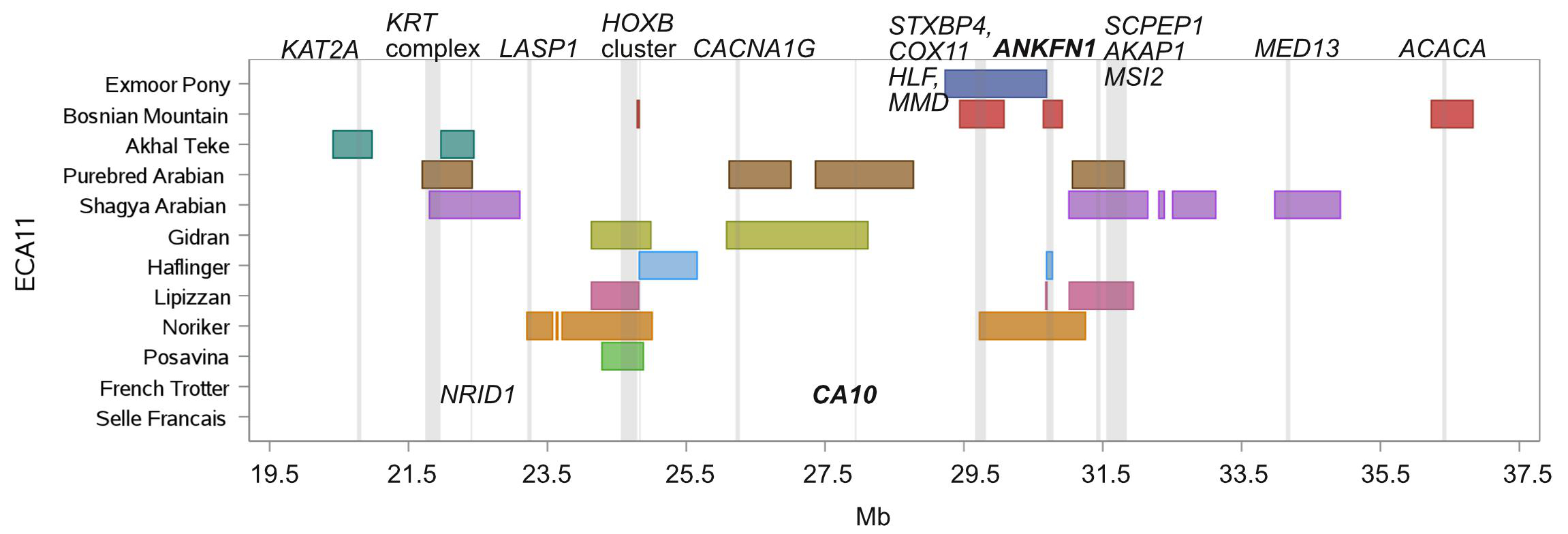


*ECA11: Plot of ROH islands (shared by more than 50% of individuals per breed) per breed including genes of specific interest.*


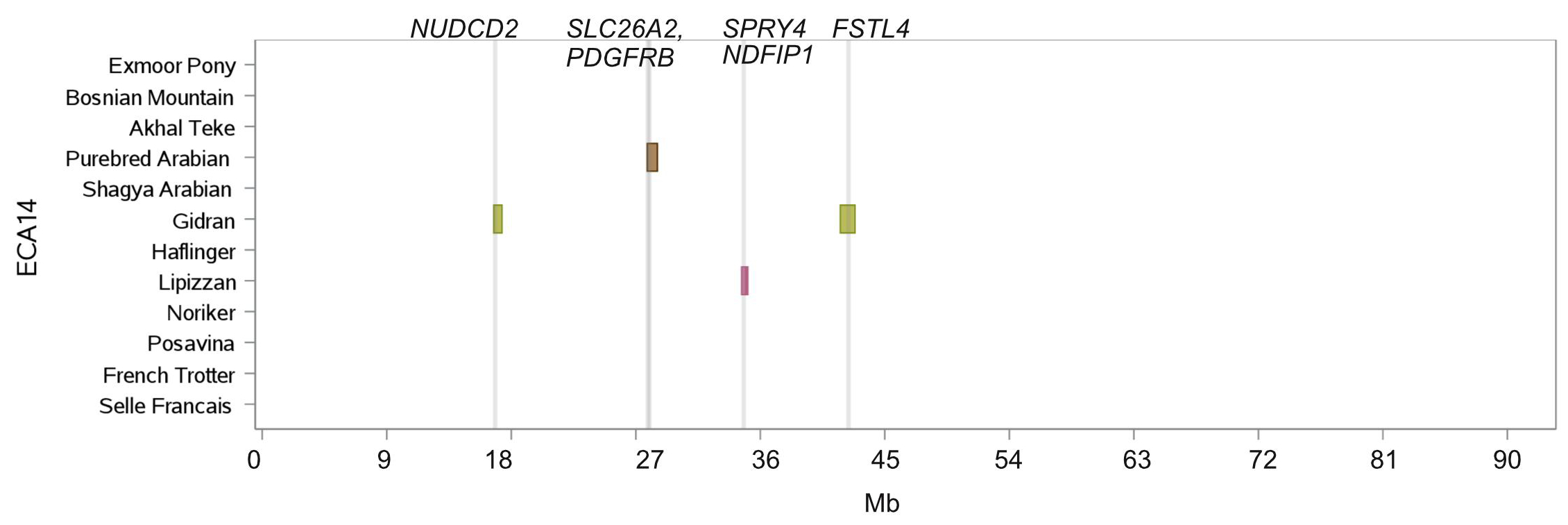


*ECA14: Plot of ROH islands (shared by more than 50% of individuals per breed) per breed including genes of specific interest.*


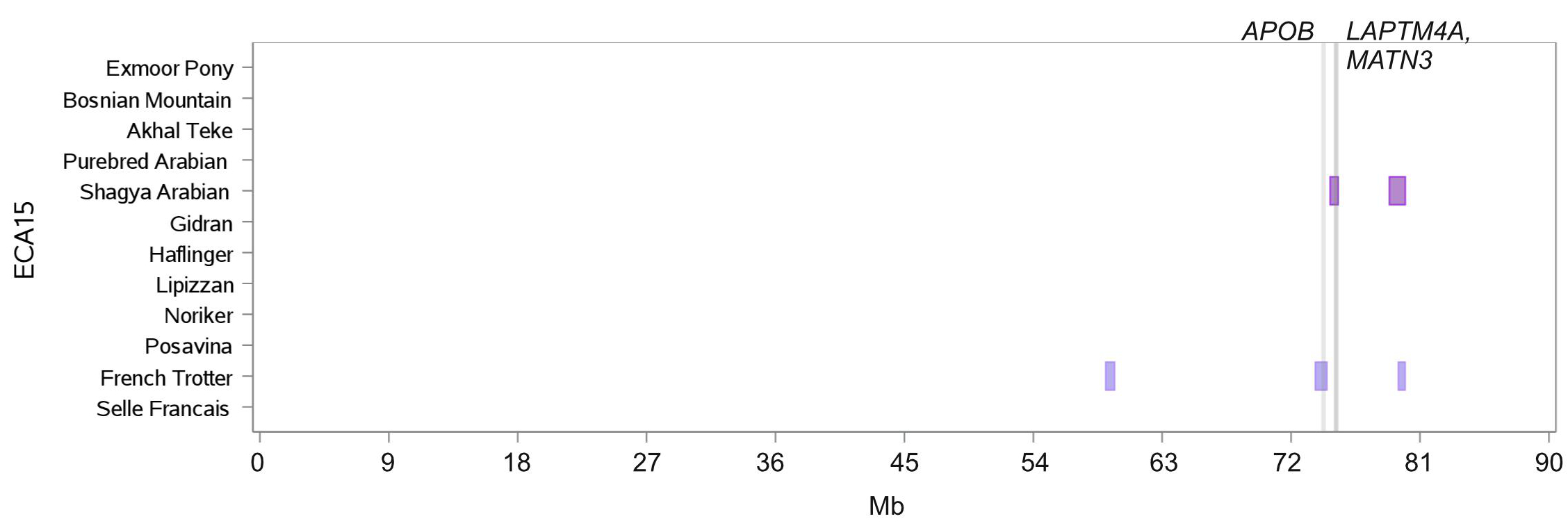


*ECA15: Plot of ROH islands (shared by more than 50% of individuals per breed) per breed including genes of specific interest.*


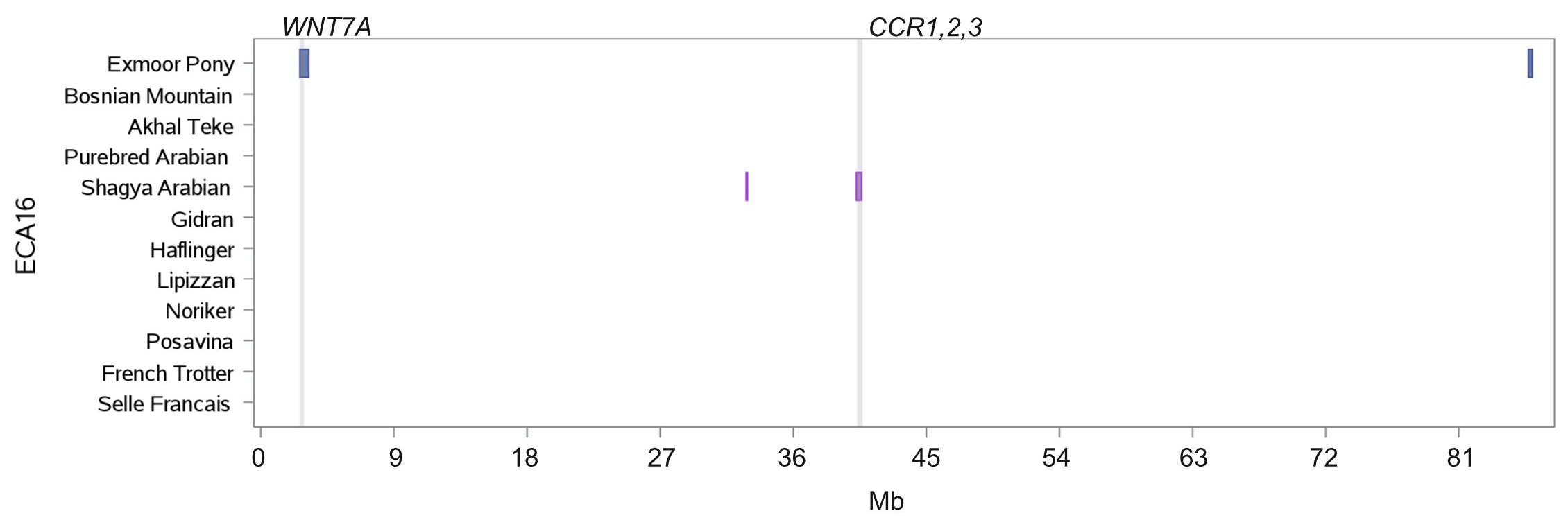


*ECA16: Plot of ROH islands (shared by more than 50% of individuals per breed) per breed including genes of specific interest.*


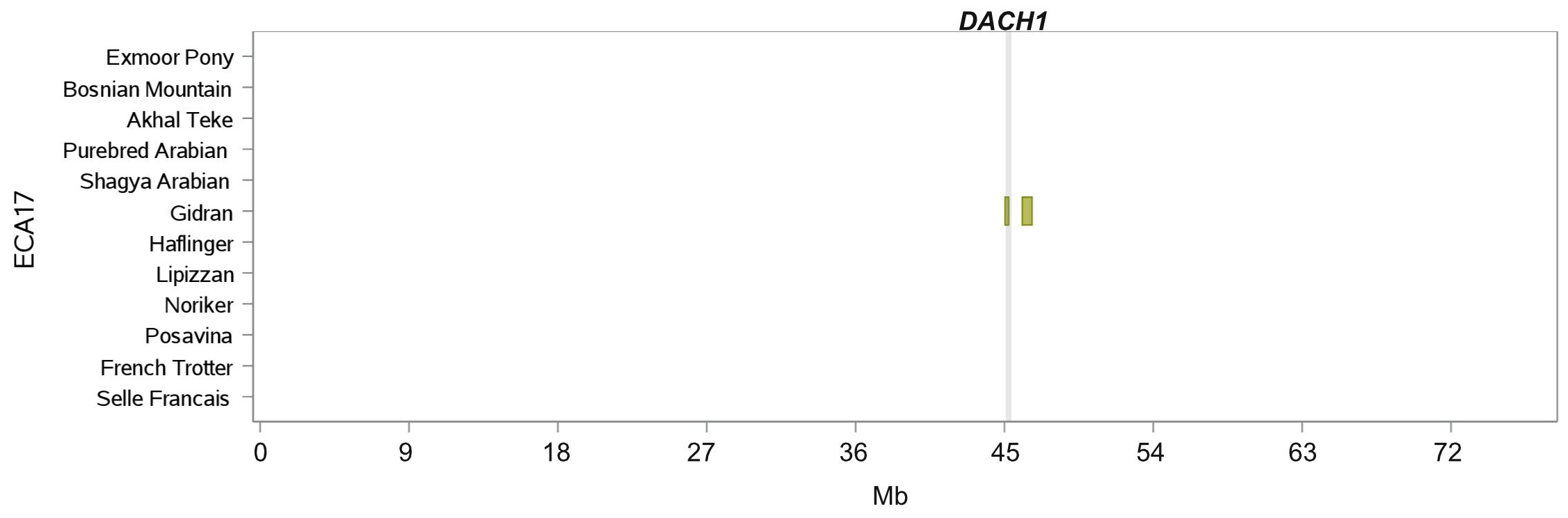


*ECA17: Plot of ROH islands (shared by more than 50% of individuals per breed) per breed including genes of specific interest.*


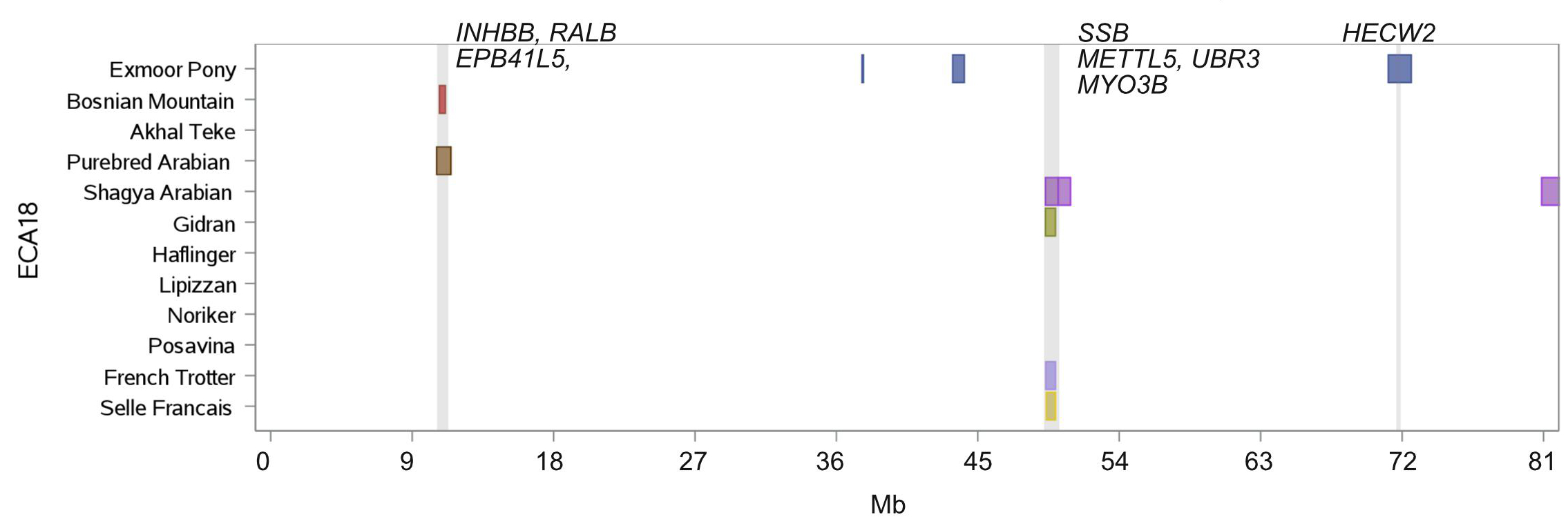


*ECA18: Plot of ROH islands (shared by more than 50% of individuals per breed) per breed including genes of specific interest.*


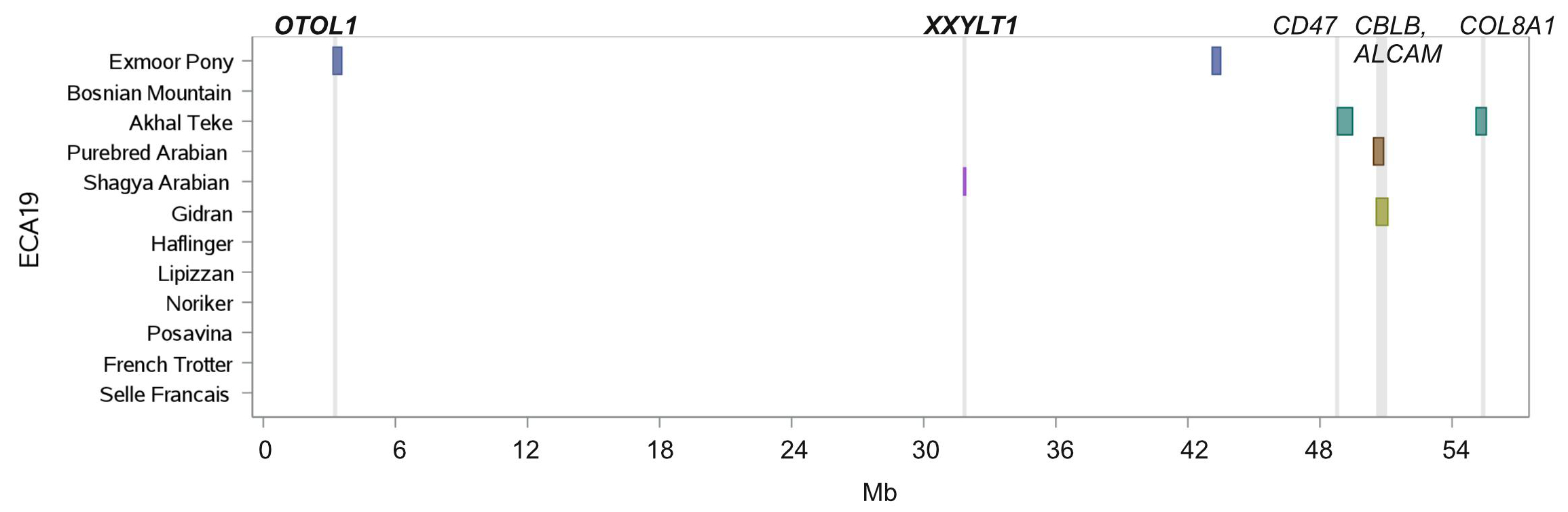


*ECA19: Plot of ROH islands (shared by more than 50% of individuals per breed) per breed including genes of specific interest.*


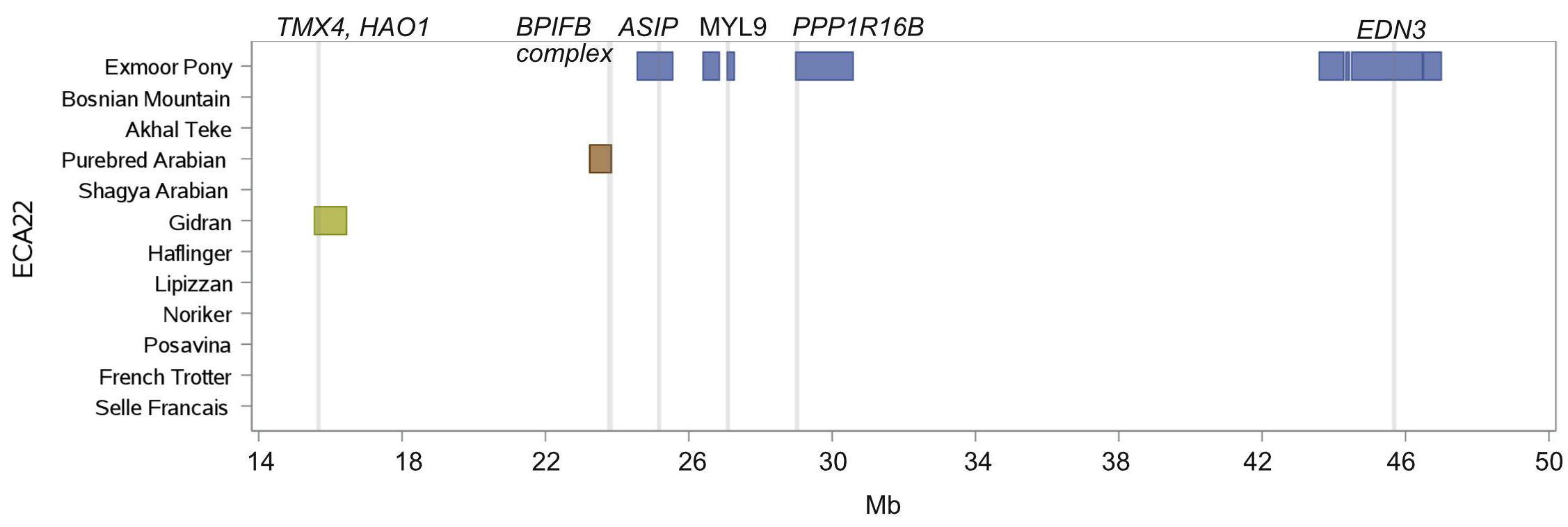


*ECA22: Plot of ROH islands (shared by more than 50% of individuals per breed) per breed including genes of specific interest.*


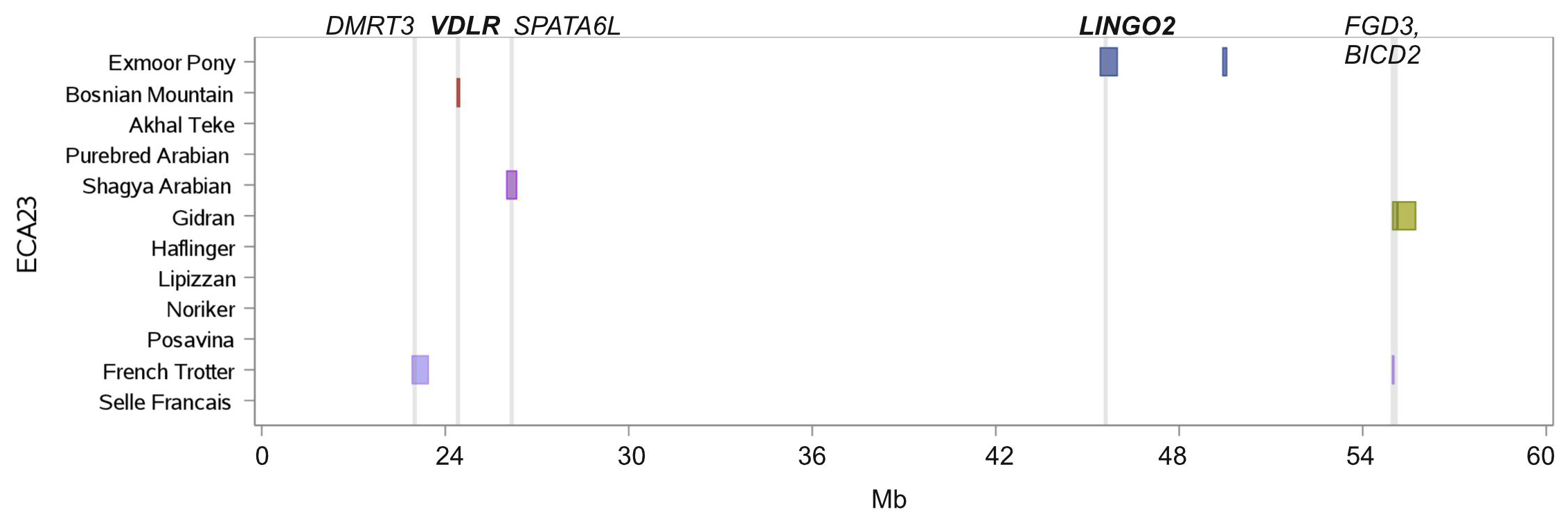


*ECA23: Plot of ROH islands (shared by more than 50% of individuals per breed) per breed including genes of specific interest.*


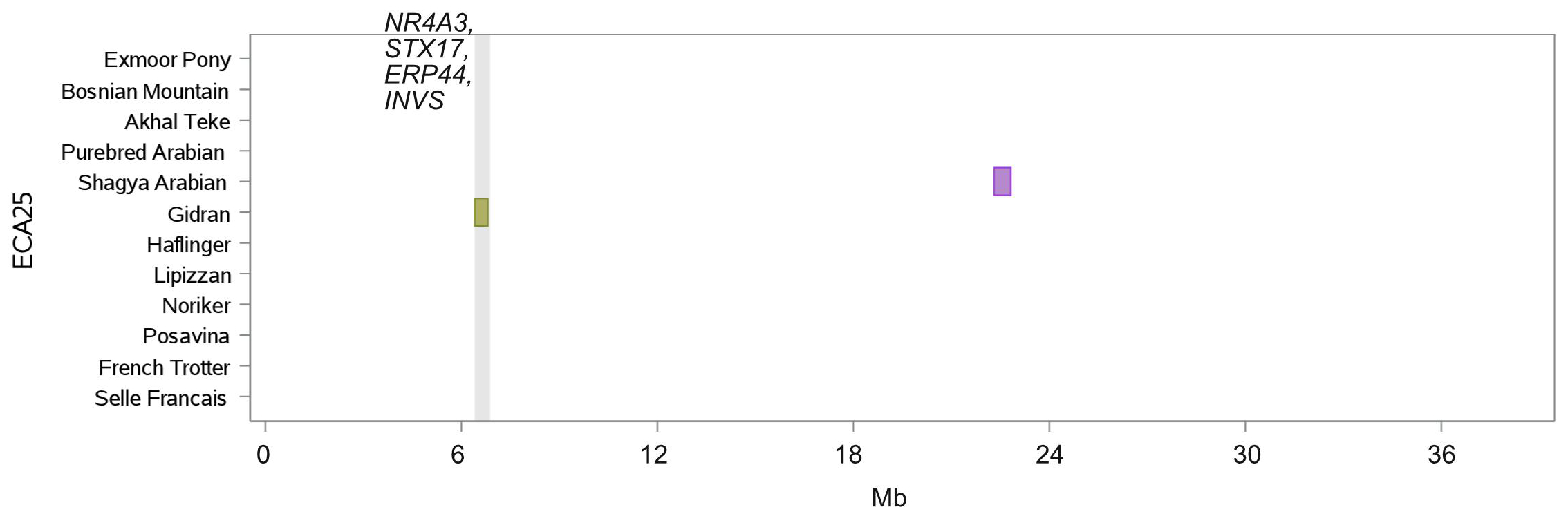


*ECA25: Plot of ROH islands (shared by more than 50% of individuals per breed) per breed including genes of specific interest.*


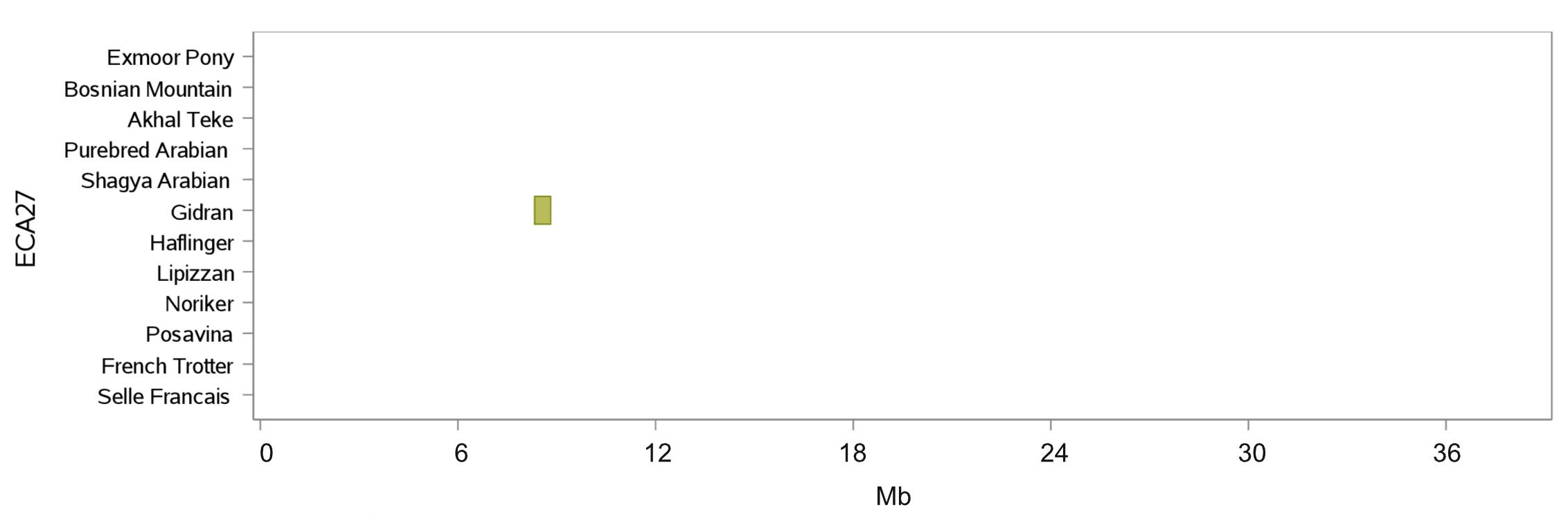


*ECA27: Plot of ROH islands (shared by more than 50% of individuals per breed) per breed including genes of specific interest.*


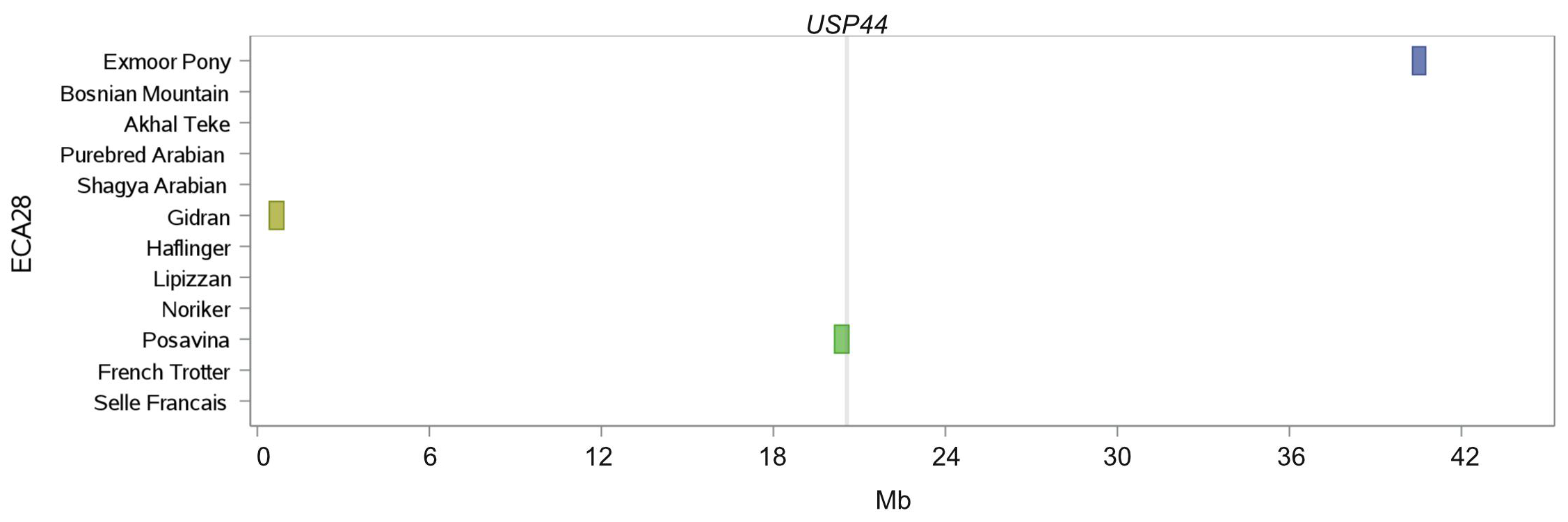


*ECA28: Plot of ROH islands (shared by more than 50% of individuals per breed) per breed including genes of specific interest.*


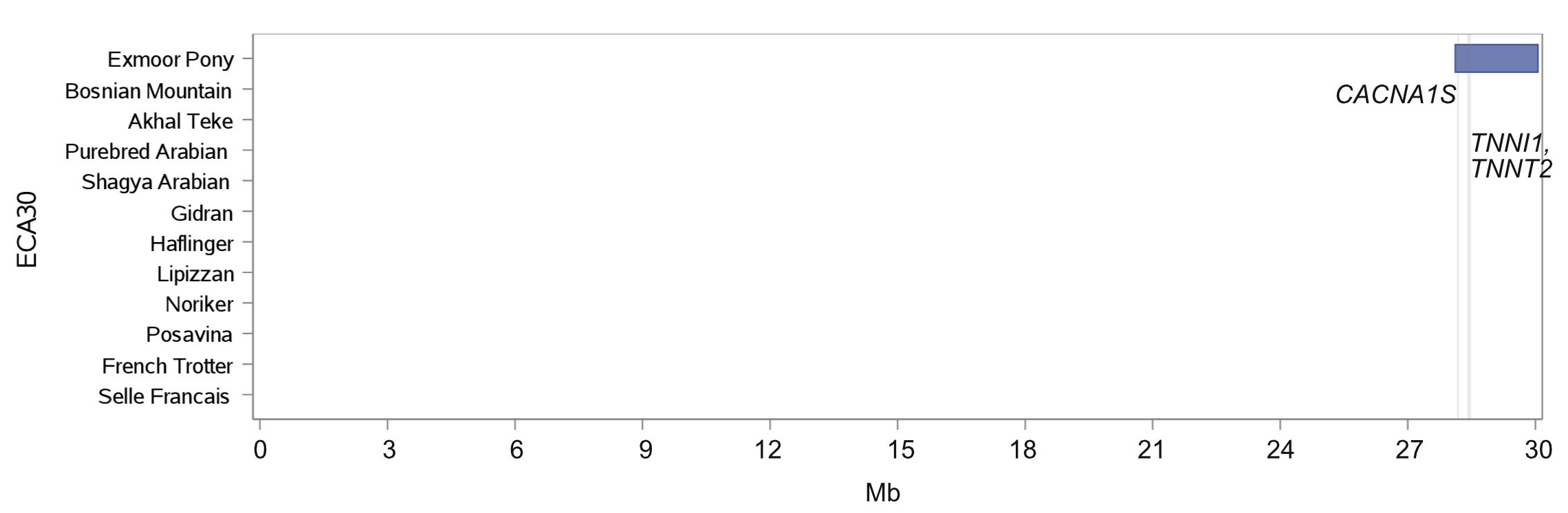


*ECA30: Plot of ROH islands (shared by more than 50% of individuals per breed) per breed including genes of specific interest.*
