# Supplementary material for: Genome-Wide Homozygosity Patterns and Evidence for Selection in a Set of European and Near Eastern Horse Breeds
Source: Genes (Basel). 2019 Jun 28;10(7):491. doi: 10.3390/genes10070491 (PMC6679042; doi:10.3390/genes10070491)

**Supplementary Figure 2.** ROH distribution on ECA7 in the breeds French Trotter (red) and Selle Francais (turqouis). On the bottom the frequency of animals sharing a ROH is visualized.


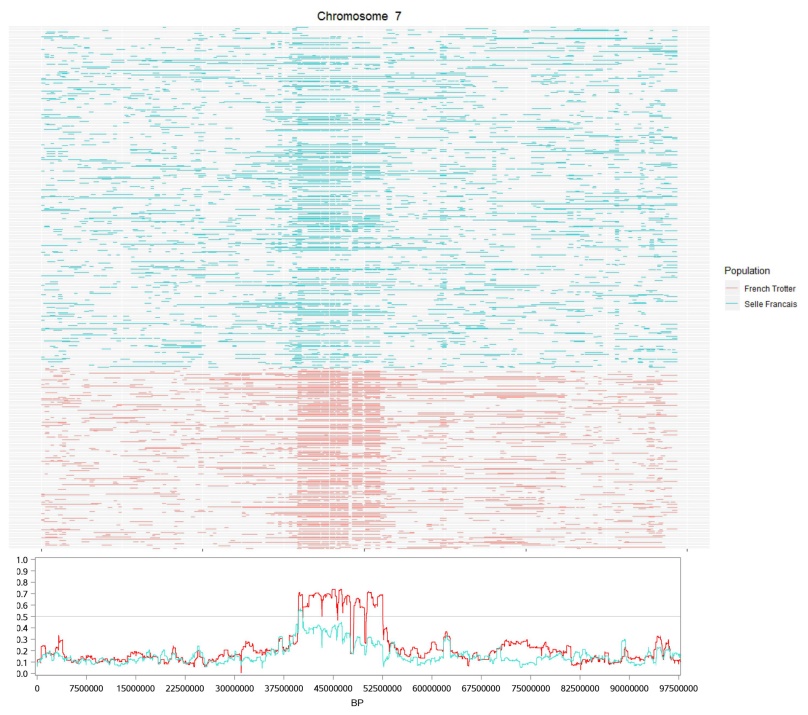

Supplement: Supplementary file 1 [file genes-10-00491-s001.zip › Supplementary_file3.docx]
