# Supplementary material for: Genome-Wide Homozygosity Patterns and Evidence for Selection in a Set of European and Near Eastern Horse Breeds
Source: Genes (Basel). 2019 Jun 28;10(7):491. doi: 10.3390/genes10070491 (PMC6679042; doi:10.3390/genes10070491)
Supplement: Supplementary file 1 [file genes-10-00491-s001.zip › supplementary_file5.docx]

**Supplementary File 5. Genotype and minor/major allele frequencies of the SNP AX-103191894 in intron 7 of the *UVSSA* gene**

Figure S1 illustrates the distribution of major/minor allele frequencies (MAF) and SNP genotype frequencies of the SNP AX-103191894 in intron 7 of the *UVSSA* gene per breed. The distributions of MAF and genotypes correspond with a geographical North-South gradient. In the Oriental group homozygosis for the reference allele *A* ranged from 0.60 to 0.78. The Lipizzan, where a quarter of the founder gene pool and the gray coat color originated from Oriental horses, reached comparable proportions. Close to this group follows the Bosnian Mountain horse, the native horse of the Balkan Peninsula. The middle European group was characterized by homozygosity levels for the *A* allele of approximately 0.30 to nearly 0.50 and contained the breeds Noriker, Haflinger, French Trotter, Selle Francais and Gidran. An inverse genotype frequency was found in the Exmoor Pony (0.9 *A/A*), and the Posavina, which has been deeply influenced by the Ardenner breed.

**Figure S1.** Distribution of minor/major allele frequencies of the SNP AX-103191894 in intron 7 of the *UVSSA* gene on the left; genotype frequencies in percent (absolute number in brackets) on the right.


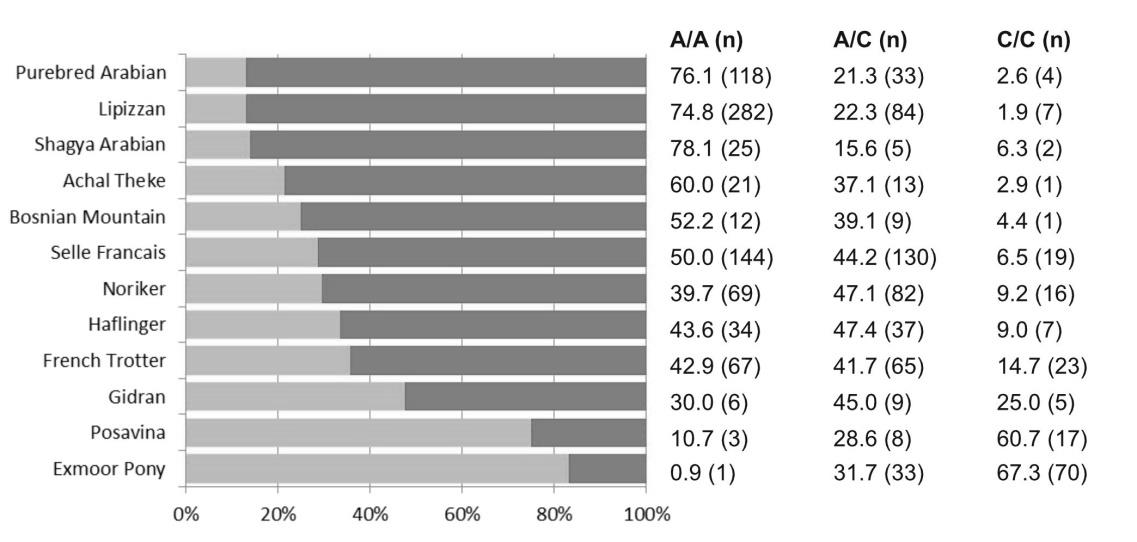


Literature:

Zechner P, Sölkner J, Bodo I, Baumung R, Achmann R, Marti E, Habe F, Brem G. 2002. Analysis of diversity and population structure in the Lipizzan horse breed based on pedigree information. *Livest Prod Sci*. 77: 137-146
