# Supplementary material for: Genome-Wide Homozygosity Patterns and Evidence for Selection in a Set of European and Near Eastern Horse Breeds
Source: Genes (Basel). 2019 Jun 28;10(7):491. doi: 10.3390/genes10070491 (PMC6679042; doi:10.3390/genes10070491)
Supplement: Supplementary file 1 [file genes-10-00491-s001.zip › supplementary_file7.docx]

**Supplementary File 7**. Gene Ontology and enrichment analysis

Gene Ontology (GO) and enrichment analysis of the French Trotter gene list

The gene list of the French trotter harboured 156 genes, 21 of them were found to be enriched in seven biological processes (BP) and three molecular functions (MF). In total 16 genes, related to the cellular component (CC) mitochondrium, and 29 genes related to the extracellular exosome, were more frequent than expected. The genes *IGFBP-1* (insulin-like growth factor-binding protein 1) and *IGFBP-3* (insulin-like growth factor-binding protein 1), were found 33,4 fold enriched and highlighted for the term regulation of insulin-like growth factor receptor signalling pathway. GO analysis outpointed eleven genes involved in positive regulation of gene expression and RNA and mRNA processing. High significance levels (p<0.0003, Bonferroni p<0.04) for enrichment were outpointed for the GO term integrin binding based upon four genes (*ICAM1, ICAM3, ICAM4, ICAM5*). Furthermore, we found an overrepresentation of genes related to the biological processes cellular response to calcium ion (*CLIC4, BRAF, JUNB*), intestinal cholesterol absorption (*LDLR, NPC1L1*) and myeloid progenitor cell differentiation (*BRAF, JAM3*).

| **Category** | **Term** | **p-value** | **Genes** | **Fold Enrichment** | **Bonferroni p-value** |
| --- | --- | --- | --- | --- | --- |
| Biological process | GO:0071277~cellular response to calcium ion | 0,017 | *BRAF, CLIC4, JUNB* | 14,59 | 0,999 |
|  | GO:0010628~positive regulation of gene expression | 0,022 | *APOB, LDLR, BRAF, DNMT1, CALR* | 4,67 | 0,999 |
|  | GO:0006397~mRNA processing | 0,028 | *SRRM1, PRKACA, HNRNPLL* | 11,30 | 0,999 |
|  | GO:0002318~myeloid progenitor cell differentiation | 0,033 | *BRAF, JAM3* | 58,36 | 1,000 |
|  | GO:0042159~lipoprotein catabolic process | 0,033 | *APOB, LDLR* | 58,36 | 1,000 |
|  | GO:0030299~intestinal cholesterol absorption | 0,033 | *LDLR, NPC1L1* | 58,36 | 1,000 |
|  | GO:0006396~RNA processing | 0,039 | *SSB, TRUB1, DHX57* | 9,46 | 1,000 |
|  | GO:0043567~regulation of insulin-like growth factor receptor signaling pathway | 0,058 | *IGFBP1, IGFBP3* | 33,35 | 1,000 |
|  |  |  |  |  |  |
| Cellular component | GO:0005739~mitochondrion | 0,002 | *GCDH, MRPL4, BRAF, RAB3D, TBRG4, PRDX2, ILF3, TRMT1, QTRT1, ECSIT, OGDH, ZMIZ2, CLIC4, PRKACA, ACAD8, DHX57* | 2,49 | 0,171 |
|  | GO:0070062~extracellular exosome | 0,005 | *ADCY1, LDHA, SLC44A2, RAB3D, OPCML, ACP5, PRDX2, CALR, CDC37, TMED4, APOB, GALM, FAM49B, DNAJC11, PRKACA, CC2D1A, YES1, ANO6, TMEM205, ICAM1, ICAM3, DNASE2, SRSF7, RAB19, CLIC4, DNAJB1, ASNA1, IGFBP3, DNM2* | 1,68 | 0,413 |
|  |  |  |  |  |  |
| Molecular function | GO:0005178~integrin binding | 0,001 | *ICAM1, ICAM4, ICAM5, ICAM3* | 28,48 | 0,045 |
|  | GO:0031994~insulin-like growth factor I binding | 0,043 | *IGFBP1, IGFBP3* | 45,57 | 0,997 |
|  | GO:0031995~insulin-like growth factor II binding | 0,043 | *IGFBP1, IGFBP3* | 45,57 | 0,997 |

Gene Ontology and enrichment analysis of the Purebred Arabian sample

In the Purebred Arabian the GO analysis revealed the terms hair follicle morphogenesis (BP) and intermediate filament (CC) for several members of the fibrous structure proteins Keratin. Intermediate filament (CC) was also assigned for the Shagya Arabian. Beside the aforementioned terms, GO analysis identified seven terms, three related to biological processes (cellular response to lipopolysaccharide, GMP metabolic process, ossification) and four to molecular functions (structural molecule activity, lipid binding, 3`,5`-cyclic-GMP phosphodiesterase activity, solute:proton antiporter activity). The KEGG pathway lysosome underlined enrichment for the genes *LAPTM4B, MFSD8, MANBA* and *IDUA*.

| **Category** | **Term** | **p-value** | **Genes** | **Fold Enrichment** | **Bonferroni p-value** |
| --- | --- | --- | --- | --- | --- |
| Biological process | GO:0031069~hair follicle morphogenesis | 0,009680 | *KRT25, KRT27, INTU* | 19,77 | 0,988822 |
|  | GO:0071222~cellular response to lipopolysaccharide | 0,011289 | *NR1D1, NFKB1, RARA, SPON2* | 8,49 | 0,994728 |
|  | GO:0046037~GMP metabolic process | 0,015741 | *PDE6A, PDE6B* | 125,24 | 0,999345 |
|  | GO:0001503~ossification | 0,021838 | *THRA, SLC26A2, PPARGC1B* | 12,96 | 0,999963 |
|  | GO:0032091~negative regulation of protein binding | 0,043359 | *WFIKKN2, EPB41L5, RALB* | 8,95 | 1,000000 |
|  |  |  |  |  |  |
| Cellular component | GO:0005882~intermediate filament | 0,000000 | *KRT26, KRT25, KRT28, KRT27, KRT12, KRT20, KRT23, KRT24* | 23,76 | 0,000005 |
|  |  |  |  |  |  |
| Molecular function | GO:0005198~structural molecule activity | 0,000153 | *KRT26, KRT25, KRT28, KRT27, KRT12, KRT20, KRT23, KRT24* | 6,86 | 0,021092 |
|  | GO:0008289~lipid binding | 0,010074 | *BPIFB2, BPIFB3, BPIFB4, BPIFB6* | 8,85 | 0,755214 |
|  | GO:0047555~3',5'-cyclic-GMP phosphodiesterase activity | 0,026627 | *PDE6A, PDE6B* | 73,72 | 0,976512 |
|  | GO:0015299~solute:proton antiporter activity | 0,043986 | *SLC9B1, SLC9B2* | 44,23 | 0,998075 |
|  |  |  |  |  |  |
| KEGG Pathway | ecb04142:Lysosome | 0,055761 | *LAPTM4B, MFSD8, MANBA, IDUA* | 4,51 | 0,999315 |

Gene Ontology and enrichment analysis of the Shagya Arabian gene list

In Shagya Arabian four out of 11 GO BP terms were related to embryogenesis: embryonic hindlimb morphogenesis, positive regulation of gastrulation, pronephros development and Type I pneumocyte differentiation. An enrichment of the genes MED1, MED13, MED18 and MED24, which are members of the mediator complex and act as transcriptional coactivators (Malik and Roeder, 2005), was highlighted in four additional GO terms. MED1 is also suggested as a coactivator for Vitamin D receptor in keratocytes and it is involved in keratocyte differentiation and proliferation (Oda et al., 2010).

| **Category** | **Term** | **p-Value** | **Genes** | **Fold Enrichment** | **Bonferroni** |
| --- | --- | --- | --- | --- | --- |
| Biological process | GO:0006935~chemotaxis | 0,008 | *CCRL2, CCR3, CCR2, PTAFR* | 9,76 | 0,994 |
|  | GO:0030521~androgen receptor signaling pathway | 0,017 | *MED24, MED13, MED1* | 14,64 | 1,000 |
|  | GO:0048822~enucleate erythrocyte development | 0,025 | *MAEA, MED1* | 78,07 | 1,000 |
|  | GO:0035116~embryonic hindlimb morphogenesis | 0,031 | *OSR2, OSR1, MED1* | 10,65 | 1,000 |
|  | GO:0006367~transcription initiation from RNA polymerase II promoter | 0,037 | *MED24, MED13, MED1* | 9,76 | 1,000 |
|  | GO:0070816~phosphorylation of RNA polymerase II C-terminal domain | 0,038 | *CDK12, GTF2H3* | 52,05 | 1,000 |
|  | GO:0036023~embryonic skeletal limb joint morphogenesis | 0,038 | *OSR2, OSR1* | 52,05 | 1,000 |
|  | GO:0048793~pronephros development | 0,038 | *OSR2, OSR1* | 52,05 | 1,000 |
|  | GO:0043066~negative regulation of apoptotic process | 0,040 | *MAEA, MTDH, OSR1, LTF, RARA, MIEN1, MED1* | 2,77 | 1,000 |
|  | GO:2000543~positive regulation of gastrulation | 0,050 | *OSR2, OSR1* | 39,04 | 1,000 |
|  | GO:0060509~Type I pneumocyte differentiation | 0,050 | *THRA, CREB1* | 39,04 | 1,000 |
| Cellular component | GO:0005882~intermediate filament | 0,000 | *KRT26, KRT25, KRT28, KRT27, TLK2, KRT24* | 12,39 | 0,019 |
|  | GO:0016592~mediator complex | 0,007 | *MED18, MED24, MED13, MED1* | 10,32 | 0,678 |
|  | GO:0010494~cytoplasmic stress granule | 0,039 | *DDX1, PUM2, GRB7* | 9,53 | 0,999 |
|  | GO:0005634~nucleus | 0,051 | *MAEA, THRA, ERBB2, SESN2, CCNE2, RPL30, OSR2, OSR1, PPP1R1B, DYNLL2, LTF, RHOB, MLXIP, TLK2, ARL6IP4, TOP2A, KMT5A, PSMD9, METTL8, CTBP1, TBX2, CREB1, TBX4, YTHDF2, FAM76A, EYA3, TRNAU1AP, TJP1, ZPBP2, CDK2AP1, NEUROD2, MPO, ST18, MED1, TP53INP1* | 1,35 | 1,000 |
| Molecular function | GO:0001104~RNA polymerase II transcription cofactor activity | 0,007 | *MED18, MED24, MED13, MED1* | 10,32 | 0,723 |
|  | GO:0005198~structural molecule activity | 0,022 | *KRT26, KRT25, KRT28, KRT27, EPB41, KRT24* | 3,72 | 0,988 |
|  | GO:0044822~poly(A) RNA binding | 0,044 | *MTDH, RBM12B, DDX1, SSB, YTHDF2, CASC3, PPP1CC, BMS1, TRNAU1AP, RPL30, DDX55, HNRNPF, POP1, MSI2, AKAP1, ARL6IP4, TOP2A* | 1,67 | 1,000 |
|  | GO:0004950~chemokine receptor activity | 0,049 | *CCRL2, CCR2* | 40,00 | 1,000 |

Gene Ontology and enrichment analysis of the Akhal Teke gene list

In the Akhal Teke sample the three GO BP terms positive regulation of T-helper 2 cell differentiation, interleukin-6-mediated signalling pathway and cellular response to lipopolysaccharide indicated enrichment of genes involved in immune defence. Further the steroid hormone receptor activity was underlined by GO analysis for the genes *THRA, NR1G1, RARA.*

| **Category** | **Term** | **p-value** | **Genes** | **Fold Enrichment** | **Bonferroni p-value** |
| --- | --- | --- | --- | --- | --- |
| Biological process | GO:0045630~positive regulation of T-helper 2 cell differentiation | 0,029 | *IL6, RARA* | 66,79 | 0,999 |
|  | GO:0070102~interleukin-6-mediated signaling pathway | 0,034 | *IL6, STAT3* | 57,25 | 0,999 |
|  | GO:0071222~cellular response to lipopolysaccharide | 0,034 | *IL6, NR1D1, RARA* | 10,19 | 0,999 |
|  | GO:0035264~multicellular organism growth | 0,038 | *KAT2A, CCM2, RARA* | 9,54 | 0,999 |
|  |  |  |  |  |  |
| Cellular component | GO:0005654~nucleoplasm | 0,010 | *COASY, CDC6, HSD17B1, BBX, ACLY, BANP, CNP, STAT3, SMARCE1, ZMIZ2, DNAJC7, ATP6V0A1, TOP2A* | 2,24 | 0,565 |
|  | GO:0000790~nuclear chromatin | 0,022 | *SMARCE1, NR1D1, RARA, STAT3* | 6,62 | 0,836 |
|  |  |  |  |  |  |
| Molecular function | GO:0031490~chromatin DNA binding | 0,014 | *THRA, RARA, STAT3* | 16,11 | 0,751 |
|  | GO:0005524~ATP binding | 0,018 | *CDC6, UBE2D4, COASY, DDX56, GCK, MYO1G, BUB1B, ACLY, CAMK2B, TOP2A, DHX58* | 2,26 | 0,828 |
|  | GO:0003707~steroid hormone receptor activity | 0,018 | *THRA, NR1D1, RARA* | 14,10 | 0,834 |
|  |  |  |  |  |  |
| KEGG pathway | ecb05152:Tuberculosis | 0,042 | *IL6, RAB5C, ATP6V0A1, CAMK2B* | 4,97 | 0,994 |

Gene Ontology and enrichment analysis of the Gidran gene list

From in total 143 genes located in ROH islands of the Gidran, 41 were found to be enriched in five biological processes, two cellular components and three molecular functions. The genes *PPARGC1A* and *NR4A3* were 45,8fold overrepresented and highlighted for positive regulation of fatty oxidation. *PPARGC1A* (peroxisome proliferator-activated receptor gamma, coactivator 1 alpha) encodes PGC-1α, which is a transcriptional coactivator that regulates genes involved in energy metabolism and mitochondrial biogenesis through expression of nuclear signalling proteins (Dominy and Piugserver, 2013). Overrepresentation of genes involved in transcription was underlined in four terms (transcription factor complex, nucleus, sequence-specific DNA binding, DNA-binding transcription factor activity). Additionally high significances (p<0.001, Bonferroni p< 0.01) were reached for two terms related to biological processes, based on the *HOXB*-cluster. GO analysis underlined terms involved in embryonic development (embryonic skeletal system morphogenesis, anterior/posterior pattern specification, rhombomere 4 development, facial nerve structural organization), transcription factor activity, and sequence-specific DNA binding. The *HOXB*-cluster was also underlined in three further breeds (Lipizzan, Noriker and Posavina; comp. Grilz-Seger et al., 2018; Grilz-Seger et al., 2019ab).

| **Category** | **Term** | **p-value** | **Genes** | **Fold Enrichment** | **Bonferroni p-value** |
| --- | --- | --- | --- | --- | --- |
| Biological process | GO:0048704~embryonic skeletal system morphogenesis | <0,001 | *HOXB3, HOXB1, HOXB2, HOXB7, HOXB8, HOXB5, HOXB6* | 25,86 | <0,001 |
|  | GO:0009952~anterior/posterior pattern specification | <0,001 | *HOXB3, HOXB1, HOXB2, HOXB7, HOXB8, HOXB5, HOXB6* | 11,96 | 0,010 |
|  | GO:0021570~rhombomere 4 development | 0,017 | *HOXB1, HOXB2* | 114,50 | 0,999 |
|  | GO:0046321~positive regulation of fatty acid oxidation | 0,042 | *NR4A3, PPARGC1A* | 45,80 | 1,000 |
|  | GO:0048671~negative regulation of collateral sprouting | 0,042 | *FSTL4, IFRD1* | 45,80 | 1,000 |
|  | |  |  |  |  |
| Cellular component | GO:0005667~transcription factor complex | 0,006 | *ATF7IP, SNF8, HOXB13, NR4A3, DACH1, HDAC9* | 4,98 | 0,559 |
|  | GO:0005634~nucleus | 0,020 | *ZNF276, RPL13, NFKB1, CBFA2T3, IRAK3, MBTD1, TCF25, USP15, IFRD1, CPNE7, PIBF1, NR4A3, DACH1, HMGA2, PPARGC1A, FOXP2, HOXB3, RERG, HOXB1, HOXB2, HOXB8, PPM1H, HOXB5, HOXB6, MAT2B, IPPK, TOB1* | 1,54 | 0,912 |
|  |  |  |  |  |  |
| Molecular function | GO:0043565~sequence-specific DNA binding | 0,019 | *HOXB1, HOXB2, HOXB7, HOXB6, HOXB13, PPARGC1A, FOXP2* | 3,27 | 0,923 |
|  | GO:0003700~transcription factor activity, sequence-specific DNA binding | 0,032 | *HOXB2, HOXB7, HOXB8, HOXB6, NFKB1, CBFA2T3, TCF25, FOXP2* | 2,60 | 0,987 |
|  | GO:0015299~solute:proton antiporter activity | 0,042 | *SLC9B1, SLC9B2* | 46,04 | 0,996 |

Gene Ontology and enrichment analysis of the Haflinger gene list

In the Haflinger GO analysis outpointed only one term (cellular response to interleukin-6, based upon the genes *PHB* and *NFKB1*) related to biological process for the Haflinger.

| **Category** | **Term** | **p-value** | **Genes** | **Fold Enrichment** | **Bonferroni p-value** |
| --- | --- | --- | --- | --- | --- |
| Biological process | GO:0071354~cellular response to interleukin-6 | 0,024 | *PHB, NFKB1* | 79,89 | 0,982 |

Gene Ontology and enrichment analysis of the Lipizzan gene list

In Lipizzans all GO terms were based upon the HOXB cluster and three out of six reached high significance levels, also at multiple levels (comp. Grilz-Seger et al., 2019b).

| **Term** | **p-value** | **Genes** | **Fold Enrichment** | **Bonferroni p-value** |
| --- | --- | --- | --- | --- |
| ***Biological process*** |  |  |  |  |
| GO:0048704~embryonic skeletal system morphogenesis | <0.001 | *HOXB3, HOXB1, HOXB2, HOXB7, HOXB8, HOXB5, HOXB6* | 159.70 | <0.001 |
| GO:0009952~anterior/posterior pattern specification | <0.001 | *HOXB3, HOXB1, HOXB2, HOXB7, HOXB8, HOXB5, HOXB6* | 73.89 | <0.001 |
| GO:0021570~rhombomere 4 development | 0.003 | *HOXB1, HOXB2* | 707.24 | 0.134 |
| GO:0021612~facial nerve structural organization | 0.011 | *HOXB1, HOXB2* | 176.81 | 0.438 |
| ***Molecular function*** |  |  |  |  |
| GO:0043565~sequence-specific DNA binding | <0.001 | *HOXB1, HOXB2, HOXB7, HOXB6, HOXB13* | 16.37 | 0.002 |
| GO:0003700~transcription factor activity, sequence-specific DNA binding | 0.007 | *HOXB2, HOXB7, HOXB8, HOXB6* | 9.10 | 0.105 |

Gene Ontology and enrichment analysis of the Bosnian Mountain Horse gene list

For the Bosnian Mountain horse GO analysis revealed significant enrichment for the genes *MMD* and *VLDLR* with biological functions in positive regulation of protein kinase activity (comp., Grilz-Seger et al., 2018).

| ***Biological process*** | ***Genes*** | ***p-value*** | **Fold Enrichment** | **Bonferroni p-value** |
| --- | --- | --- | --- | --- |
| GO:0045860~positive regulation of protein kinase activity | *MMD, VLDLR* | 0,039 | 47,52 | 0,995 |

Gene Ontology and enrichment analysis of the Noriker gene list

In Noriker horse similar GO terms based upon the HOXB cluster as in Lipizzans were highlighted and additional BP term (angiogenesis) and the two cell compartments autophagosom and nucleoplasm were underlined (comp., Grilz-Seger et al., 2019a).

| **Category** | **Term** | **p-value** | **Genes** | **Fold Enrichment** | **Bonferroni p-value** |
| --- | --- | --- | --- | --- | --- |
| Biological process | GO:0048704~embryonic skeletal system morphogenesis | <0.001 | *HOXB3, HOXB1, PCGF2, HOXB2, HOXB7, HOXB8, HOXB5, HOXB6* | 91.26 | <0.001 |
|  | GO:0009952~anterior/posterior pattern specification | <0.001 | *HOXB3, HOXB1, PCGF2, HOXB2, HOXB7, HOXB8, HOXB5, HOXB6* | 42.22 | <0.001 |
|  | GO:0021570~rhombomere 4 development | 0.006 | *HOXB1, HOXB2* | 353.62 | 0.508 |
|  | GO:0021612~facial nerve structural organization | 0.022 | *HOXB1, HOXB2* | 88.40 | 0.941 |
|  | GO:0001525~angiogenesis | 0.029 | *HOXB3, HOXB13, TMEM100* | 10.94 | 0.977 |
| Cellular component | GO:0005776~autophagosome | 0.004 | *OSBPL7, CALCOCO2, PIP4K2B* | 31.45 | 0.162 |
|  | GO:0005654~nucleoplasm | 0.010 | *CWC25, MRPL10, HOXB7, PSMB3, SNF8, PNPO, HOXB13, KPNB1, PIP4K2B* | 2.83 | 0.358 |
| Molecular function | GO:0043565~sequence-specific DNA binding | 0.003 | *HOXB1, HOXB2, HOXB7, HOXB6, HOXB13* | 7.64 | 0.113 |
|  | GO:0003700~transcription factor activity, sequence-specific DNA binding | 0.012 | *HOXB2, HOXB7, HOXB8, HOXB6, ZFAT* | 5.31 | 0.349 |

Gene Ontology and enrichment analysis of the Posavina gene list

All GO terms in the Posavina were based upon the HOXB cluster. High significance levels (Bonferroni adjusted p-value<0.007) were reached for the GO terms anterior/posterior pattern specification (GO:0009952), embryonic skeletal system morphogenesis (GO:0048704), rhombomere 4 development (GO:0021570) and sequence-specific DNA binding (GO:0043565).

|  | **p-value** | **Genes** | **Fold Enrichment** | **Bonferroni p-value** |
| --- | --- | --- | --- | --- |
| ***Biological process*** |  |  |  |  |
| GO:0048704~embryonic skeletal system morphogenesis | 0,000000 | *HOXB3, HOXB1, HOXB2, HOXB7, HOXB8, HOXB5, HOXB6* | 135,74 | 0,000000 |
| GO:0009952~anterior/posterior pattern specification | 0,000000 | *HOXB3, HOXB1, HOXB2, HOXB7, HOXB8, HOXB5, HOXB6* | 62,81 | 0,000000 |
| GO:0021570~rhombomere 4 development | 0,003158 | *HOXB1, HOXB2* | 601,15 | 0,064270 |
| GO:0021612~facial nerve structural organization | 0,012576 | *HOXB1, HOXB2* | 150,29 | 0,180724 |
|  |  |  |  |  |
| ***Molecular function*** |  |  |  |  |
| GO:0043565~sequence-specific DNA binding | 0,000327 | *HOXB1, HOXB2, HOXB7, HOXB6, HOXB13* | 13,49 | 0,007167 |
| GO:0003700~transcription factor activity, sequence-specific DNA binding | 0,012668 | *HOXB2, HOXB7, HOXB8, HOXB6* | 7,50 | 0,130847 |
|  |  |  |  |  |

Gene Ontology and enrichment analysis of the Exmoor Pony gene list

From a total of 171 genes located in Exmoor Pony specific islands, Gene Ontology and enrichment analysis highlighted three terms (epithelial cell differentiation, cellular response to DNA stimulus, uterus morphogenesis) related to biological processes, the cellular component term troponin complex and four terms related to molecular functions (MF). Additionally four KEGG pathways (renin secretion, cGMP-PKG signaling pathway, and oxytocin signalling) were highlighted.

| **Category** | **Term** | **p-value** | **Genes** | **Fold Enrichment** | **Bonferroni p-value** |
| --- | --- | --- | --- | --- | --- |
| Biological process | GO:0030855~epithelial cell differentiation | 0,007 | *ELF3, CTSB, BMP7, CBFA2T2* | 10,12 | 0,971 |
|  | GO:0006974~cellular response to DNA damage stimulus | 0,029 | *HELB, STXBP4, HERC2, UBE2T* | 5,94 | 1,000 |
|  | GO:0061038~uterus morphogenesis | 0,044 | *KDM5B, WNT7A* | 44,53 | 1,000 |
|  | GO:0042438~melanin biosynthetic process | 0,061 | *ASIP, OCA2* | 31,81 | 1,000 |
|  |  |  |  |  |  |
| Cellular component | GO:0005861~troponin complex | 0,073 | *TNNT2, TNNI1* | 26,05 | 0,999 |
|  |  |  |  |  |  |
| Molecular function | GO:0003677~DNA binding | 0,032 | *ZNF341, POLR2K, TGIF2, MYOG, SPO11, HMGA2, KDM5B, EEPD1, ZBP1* | 2,40 | 0,984 |
|  | GO:0008599~protein phosphatase type 1 regulator activity | 0,041 | *PPP1R16B, PHACTR3* | 47,00 | 0,995 |
|  | GO:0016874~ligase activity | 0,051 | *HECW2, HERC2, ITCH* | 8,20 | 0,998 |
|  |  |  |  |  |  |
| KEGG pathway | ecb04924:Renin secretion | 0,007 | *CTSB, CACNA1S, ADORA1, ITPR2* | 9,93 | 0,587 |
|  | ecb04921:Oxytocin signaling pathway | 0,011 | *KRAS, PPP1R12B, CACNA1S, MYL9, ITPR2* | 5,56 | 0,757 |
|  | ecb04022:cGMP-PKG signaling pathway | 0,014 | *GATA4, CACNA1S, ADORA1, MYL9, ITPR2* | 5,17 | 0,837 |
|  | ecb04270:Vascular smooth muscle contraction | 0,032 | *PPP1R12B, CACNA1S, MYL9, ITPR2* | 5,61 | 0,983 |

Due to the low number and short length of ROH islands in Selle Francais, the gene list of this breed contained only eight genes and GO analysis revealed no enrichment.

**References:**

[Dominy](https://www.ncbi.nlm.nih.gov/pubmed/?term=Dominy%20JE%5BAuthor%5D&cauthor=true&cauthor_uid=23818499) JE and [Puigserver](https://www.ncbi.nlm.nih.gov/pubmed/?term=Puigserver%20P%5BAuthor%5D&cauthor=true&cauthor_uid=23818499) P. 2013. Mitochondrial biogenesis through activation of nuclear signaling proteins [cold spring harb. *Perspect Biol*](https://www.ncbi.nlm.nih.gov/pmc/articles/PMC3685894/). 5: a015008.

Grilz-Seger G, Druml T, Neuditschko M, Dobretsberger M, Horna M, Brem G. 2019b. High-resolution population structure and runs of homozygosity reveal the genetic architecture of complex traits in the Lipizzan horse. BMC Genom. 20:174.

Grilz-Seger G, Druml T, Neuditschko M, Mesarič M, Cotman M, Brem G. 2019a. Analysis of ROH patterns in the Noriker horse breed reveal signatures of selection for coat color and body size. Accepted, in production, Anim Genet.

Grilz-Seger G, Mesarič M, Cotman M, Neuditschko M, Druml T, Brem G. 2018. Runs of Homozygosity and Population History of Three Horse Breeds With Small Population Size. J Equ Vet Sci. 71: 27-34.

Malik S and Roeder RG. 2005. Dynamic regulation of pol II transcription by the mammalian Mediator complex, Trends in Biochem Sci. 30: 256-263.

Oda Y, Chalkley RJ, Burlingame AL ,Bikle DD. 2010. The transcriptional coactivator DRIP/Mediator Complex is involved in vitamin D receptor function and regulates keratinocyte proliferation and differentiation. J Investig Dermatol. 130: 2377-2388.
