# Supplementary material for: Genome-Wide Homozygosity Patterns and Evidence for Selection in a Set of European and Near Eastern Horse Breeds
Source: Genes (Basel). 2019 Jun 28;10(7):491. doi: 10.3390/genes10070491 (PMC6679042; doi:10.3390/genes10070491)
Supplement: Supplementary file 1 [file genes-10-00491-s001.zip › Supplementary_file1.doc]

Supplementary File 1: ROH islands per breed

Table S1. ROH islands of the Shagya Arabian

| **Chr.** | **Begin** | **End** | **Length (kb)** | **ROH freq.** | **Number of annotated genes** |
| --- | --- | --- | --- | --- | --- |
| 1 | 70,743.767 | 71,554.797 | 811.0 | 0.605 | 5 |
| 1 | 106,785.362 | 107,475.459 | 690.1 | 0,597 | 3 |
| 2 | 26,946.303 | 28,340.373 | 1394.1 | 0.565 | 26 |
| 2 | 69,265.949 | 69,789.558 | 532.6 | 0.642 | 4 |
| 3 | 118,525.423 | 119,479.623 | 954.2 | 0.735 | 17 |
| 4 | 50,883.038 | 50,904.590 | 21.6 | 0.531 | 1 |
| 6 | 57,520.291 | 57,544.118 | 23.8 | 0.531 | - |
| 7 | 39,560.127 | 41,127.737 | 1557,6 | 0.659 | 2 |
| 8 | 21,211.062 | 21,221.889 | 10.8 | 0.531 | 1 |
| 8 | 22,133.856 | 22,621.489 | 487.6 | 0.531 | 13 |
| 8 | 22,775.975 | 23,659.576 | 883.6 | 0.533 | 24 |
| 9 | 30,594.323 | 32,349.209 | 1754.9 | 0.618 | 5 |
| 9 | 38,858.048 | 40,334.842 | 1476.8 | 0.565 | 3 |
| 9 | 41,132.507 | 41,512.607 | 380.1 | 0.557 | 5 |
| 9 | 43,851.526 | 45,695.366 | 1843.8 | 0.557 | 10 |
| 9 | 50,300.138 | 50,330.433 | 30.3 | 0.531 | - |
| 9 | 77,634.541 | 78,249.446 | 614.9 | 0.559 | 2 |
| 11 | 21,801.064 | 23,102.984 | 1301.9 | 0,652 | 42 |
| 11 | 31,012.276 | 32,149.204 | 1136.9 | 0.708 | 7 |
| 11 | 32,307.957 | 32,385.588 | 77.6 | 0.576 | 1 |
| 11 | 32,505.269 | 33,127.954 | 622.7 | 0.651 | 10 |
| 11 | 33,977.606 | 34,921.749 | 944.1 | 0.594 | 5 |
| 15 | 74,724.754 | 75,283.566 | 558.8 | 0.613 | 7 |
| 15 | 78, 860.607 | 79,968.808 | 1108.2 | 0.595 | 2 |
| 16 | 32,816.611 | 32,851.808 | 35.2 | 0.557 | - |
| 16 | 40,263.652 | 40,615.958 | 352.3 | 0.513 | 5 |
| 18 | 49,306.143 | 50,102.739 | 796.6 | 0.708 | 4 |
| 18 | 50,114.593 | 50,895.764 | 781.2 | 0.571 | 7 |
| 18 | 80,882.137 | 82,130.273 | 1248.1 | 0.565 | 10 |
| 19 | 31,813.838 | 31,904.262 | 90.4 | 0.531 | 1 |
| 23 | 26,014.258 | 26,329.242 | 314.9 | 0.561 | 5 |
| 25 | 22,304.452 | 22,816.590 | 512.1 | 0.598 | - |

Table S2. ROH islands of the Purebred Arabian

| **Chr.** | **Begin** | **End** | **Length (kb)** | **ROH freq.** | **Number of annotated genes** |
| --- | --- | --- | --- | --- | --- |
| 2 | 100,516.831 | 101,658.037 | 1,141.1 | 0.561 | 6 |
| 3 | 35,894.802 | 35,922.591 | 27.7 | 0.503 | - |
| 3 | 35,947.834 | 35976.199 | 28.3 | 0.503 | 2 |
| 3 | 36,047.262 | 38,171.065 | 2,123.803 | 0.675 | 20 |
| 3 | 118,659.800 | 119,479.758 | 819.9 | 0.714 | 16 |
| 4 | 54,926.177 | 55,797.447 | 871.2 | 0.593 | 6 |
| 5 | 52,342.712 | 53,122.526 | 779.8 | 0.548 | 5 |
| 7 | 40,068.677 | 41,337.465 | 1,268.7 | 0.567 | 2 |
| 7 | 50,167.155 | 50,932.454 | 765.2 | 1.000 | 1 |
| 9 | 31,329.939 | 32,034.084 | 704.1 | 0.531 | 2 |
| 9 | 44,001.641 | 44,562.960 | 561.3 | 0.572 | 5 |
| 11 | 21,697.011 | 22,416.682 | 722.1 | 0.626 | 21 |
| 11 | 26,117.473 | 27,008.802 | 891.3 | 0.504 | 17 |
| 11 | 27,363.048 | 28,771.694 | 1,408.6 | 0.515 | 1 |
| 11 | 31,062.702 | 31,810.316 | 747.6 | 0.622 | 6 |
| 14 | 27,834.373 | 28,556.515 | 722.1 | 0.507 | 13 |
| 18 | 10,561.182 | 11,460565 | 899.3 | 0.567 | 4 |
| 19 | 50,436.512 | 50,890.075 | 453.5 | 0.574 | 2 |
| 22 | 23,234.629 | 23,837.810 | 603.1 | 0.554 | 11 |

Table S3. ROH islands of the Gidran

| **Chr.** | **Begin** | **End** | **Length (kb)** | **ROH freq.** | **Number of annotated genes** |
| --- | --- | --- | --- | --- | --- |
| 2 | 105,497.161 | 105,796.864 | 229.7 | 0.595 | 4 |
| 2 | 116,836.509 | 117,094.945 | 258.4 | 0.553 | 2 |
| 3 | 35,408.432 | 36,013.053 | 739.5 | 0.562 | 18 |
| 3 | 36,890.729 | 38,237.104 | 1346.4 | 0.871 | 9 |
| 3 | 100,730.927 | 101,174.966 | 444.0 | 0.509 | 1 |
| 4 | 36,389.037 | 36,494.123 | 105.1 | 0.550 | 1 |
| 4 | 50,539.528 | 51,104.180 | 562.7 | 0.556 | 1 |
| 4 | 52,093.254 | 53,089.373 | 996.1 | 0.553 | 2 |
| 4 | 70,050.031 | 70,099.414 | 49.4 | 0.550 | - |
| 4 | 70,269.531 | 70,784.979 | 515.4 | 0.550 | 5 |
| 4 | 71,907.978 | 72,106.140 | 198.16 | 0.550 | 1 |
| 4 | 72,461.834 | 72,477.963 | 16.13 | 0.550 | - |
| 6 | 41,121.985 | 42,740.210 | 16182.6 | 0.70 | 13 |
| 6 | 78,594.591 | 79,196.095 | 601.5 | 0.551 | 3 |
| 6 | 81,156.975 | 81,795.964 | 639.0 | 0.799 | 3 |
| 9 | 69,400.484 | 69,991.652 | 591.2 | 0.620 | - |
| 11 | 24,134.125 | 24,990.996 | 856.9 | 0.68 | 17 |
| 11 | 26,082.230 | 28,118.531 | 2036.3 | 0.503 | 19 |
| 14 | 16,740.416 | 17,331.411 | 590.9 | 0.568 | 4 |
| 14 | 41,779.891 | 42,846.696 | 1066.8 | 0.591 | 9 |
| 17 | 45,034.791 | 45,261.930 | 227.1 | 0.550 | 1 |
| 17 | 46,080.450 | 46,665.203 | 584.8 | 0.672 | 4 |
| 18 | 49,300.808 | 49,940.945 | 640.1 | 0.584 | 4 |
| 19 | 50,554.541 | 51,079.622 | 933.9 | 0.573 | 2 |
| 22 | 15,554.541 | 16,448.501 | 893.9 | 0.777 | 2 |
| 23 | 54,984.719 | 55,118.871 | 134.2 | 0.502 | 2 |
| 23 | 55,152.569 | 55,725.351 | 572.8 | 0.543 | 8 |
| 25 | 6,409.342 | 6,812.785 | 403.4 | 0.550 | 4 |
| 27 | 8,325.935 | 8,814.195 | 488.3 | 0.550 | - |
| 28 | 416.364 | 924.561 | 918.3 | 0.550 | 4 |

Table S4. ROH islands of the Akhal Teke

| **Chr.** | **Begin** | **End** | **Length (kb)** | **ROH freq.** | **Number of annotated genes** |
| --- | --- | --- | --- | --- | --- |
| 1 | 147,980.894 | 148,516.795 | 535.9 | 0.539 | 11 |
| 1 | 149,184.066 | 149,368.006 | 184.1 | 0.517 | - |
| 3 | 34,386.385 | 34,736.351 | 349.9 | 0.516 | 4 |
| 4 | 14,849.942 | 15,817.917 | 967.9 | 0.611 | 22 |
| 4 | 54,017.932 | 54,620.035 | 611.1 | 0.537 | 4 |
| 11 | 20,413.024 | 20,973.188 | 560.1 | 0.528 | 25 |
| 11 | 21,966.387 | 22,440.120 | 473.7 | 0.566 | 13 |
| 19 | 48,794.907 | 49,476.538 | 681.6 | 0.611 | 3 |
| 19 | 55,087.293 | 55,553.410 | 466.1 | 0.485 | 2 |

Table S5. ROH islands of the Noriker

| **Chr.** | **Begin** | **End** | **Length (kb)** | **ROH freq.** | **Number of annotated genes** |
| --- | --- | --- | --- | --- | --- |
| 3 | 104,695.238 | 105,832.553 | 1,137.3 | 0.61 | 3 |
| 9 | 74,971.396 | 75,661.910 | 690.5 | 0.61 | 1 |
| 11 | 23,203.615 | 23,575.186 | 371.6 | 0.53 | 10 |
| 11 | 23,712.507 | 25,010.061 | 1,297.6 | 0.58 | 29 |
| 11 | 29,724.170 | 31,250.934 | 1,526.8 | 0.66 | 7 |

Table S6. ROH islands of the Posavina

| **Chr.** | **Begin** | **End** | **Length (kb)** | **ROH freq.** | **Number of annotated genes** |
| --- | --- | --- | --- | --- | --- |
| 6 | 29,104.545 | 30,128.569 | 1,024.0 | 0.571 | 6 |
| 8 | 36,472.293 | 36,512.830 | 40.5 | 0.536 | - |
| 9 | 75,354.137 | 75,434.658 | 80.5 | 0.507 | - |
| 11 | 24,285.730 | 24,881.245 | 595.5 | 0.464 | 10 |
| 28 | 20,136.585 | 20,633.641 | 497.1 | 0.500 | 6 |

Table S7. ROH islands of the Bosnian Mountain Horse

| **Chr.** | **Begin** | **End** | **Length (kp)** | **ROH freq.** | **Number of annotated genes** |
| --- | --- | --- | --- | --- | --- |
| 1 | 148,516.795 | 148,639.327 | 122.5 | 0.522 | 4 |
| 3 | 29,712.722 | 30,371.502 | 658.8 | 0.609 | 2 |
| 6 | 29,539.506 | 30,128.928 | 589.4 | 0.522 | 5 |
| 6 | 27,249.667 | 28,055.127 | 805.5 | 0.60,9 | 7 |
| 11 | 24,793.573 | 24,822.752 | 29.2 | 0.522 | 2 |
| 11 | 29,440.642 | 30,078.455 | 637.8 | 0.700 | 4 |
| 11 | 30,645.281 | 30,915.992 | 270.7 | 0.522 | 1 |
| 11 | 36,233.441 | 36,833.929 | 600.5 | 0.520 | 7 |
| 18 | 10,733.279 | 11,116.742 | 383.5 | 0.522 | 3 |
| 23 | 24,390.937 | 24,463.864 | 72.9 | 0.520 | 1 |

Table S8. ROH islands of the Haflinger

| **Chr.** | **Begin** | **End** | **Length (kb)** | **ROH freq.** | **Number of annotated genes** |
| --- | --- | --- | --- | --- | --- |
| 3 | 34,280.529 | 36,532.152 | 2,251.6 | 0.714 | 29 |
| 3 | 37,179.496 | 38,127.255 | 947.8 | 0.545 | 4 |
| 7 | 48,157.328 | 48,930.905 | 773.5 | 0.623 | 9 |
| 11 | 24,822.752 | 25,657.958 | 835.2 | 0.525 | 17 |
| 11 | 30,690.860 | 30,774.494 | 83.6 | 0.487 | 1 |

Table S9. ROH islands of the Lipizzan

| **Chr.** | **Begin** | **End** | **Length (kb)** | **ROH freq.** | **Number of annotated genes** |
| --- | --- | --- | --- | --- | --- |
| 11 | 24,134.125 | 24,816.254 | 682.1 | 0.613 | 12 |
| 11 | 30,677.562 | 30,685.590 | 8.0 | 0.501 | - |
| 11 | 31,015.821 | 31,942.748 | 926.9 | 0.666 | 6 |
| 14 | 34,988.691 | 34,999.535 | 10.8 | 0.501 | - |
| 14 | 34,651.534 | 34,935.341 | 283.8 | 0.514 | 2 |

Table S10. ROH islands of the Selle Francais

| **Chr.** | **Begin** | **End** | **Length (kb)** | **ROH freq.** | **Number of annotated genes** |
| --- | --- | --- | --- | --- | --- |
| 1 | 84,342.144 | 84,700.700 | 358.5 | 0.522 | 1 |
| 6 | 29,424.261 | 29,462.523 | 38.2 | 0.501 | 1 |
| 6 | 29,537.838 | 30,040.425 | 502.5 | 0.522 | 3 |
| 7 | 39,713.550 | 40,333.201 | 619.6 | 0.544 | - |
| 18 | 49,355.247 | 49,940.945 | 585.6 | 0.514 | 3 |

Table S11. ROH islands of the French Trotter

| **Chr.** | **Begin** | **End** | **Length (kb)** | **ROH freq.** | **Number of annotated genes** |
| --- | --- | --- | --- | --- | --- |
| 1 | 16,326.815 | 16,983.124 | 656.3 | 0.518 | 2 |
| 2 | 30,318.999 | 30,838.488 | 519.4 | 0.567 | 7 |
| 2 | 38,309.204 | 38,470.791 | 161.5 | 0.507 | - |
| 2 | 43,216.194 | 44,018.636 | 802.4 | 0.678 | 2 |
| 4 | 15,163.634 | 16,386.121 | 1,222.5 | 0.551 | 13 |
| 4 | 52,692.165 | 52,840.002 | 147.8 | 0.506 | 1 |
| 4 | 93,416.564 | 93,872.804 | 456.2 | 0.551 | 7 |
| 6 | 41,240.508 | 41,843.164 | 602.6 | 0.531 | 1 |
| 6 | 63,568.169 | 64,142.141 | 573.7 | 0.587 | 3 |
| 7 | 39,687.420 | 40,366.168 | 678.7 | 0.677 | - |
| 7 | 40,396.269 | 45,609.959 | 5,269.7 | 0.667 | 53 |
| 7 | 48,155.562 | 49,776.609 | 1,621.0 | 0.613 | 41 |
| 7 | 50,011.398 | 52,530.827 | 2,519.4 | 0.657 | 3 |
| 8 | 32,270.266 | 33,386.931 | 1,116.5 | 0.614 | 1 |
| 8 | 41,232.707 | 41,865.448 | 632.7 | 0.554 | 3 |
| 9 | 71,079.972 | 71,580.576 | 500.6 | 0.629 | 2 |
| 15 | 59,057.669 | 59,675.524 | 617.8 | 0.596 | 7 |
| 15 | 73,695.090 | 74,493.866 | 798.7 | 0.578 | 2 |
| 15 | 79,489.871 | 79,964.006 | 474.1 | 0.518 | - |
| 18 | 49,323.427 | 49,940.945 | 617.5 | 0.579 | 4 |
| 23 | 22,921.414 | 23,440.627 | 519.2 | 0.574 | - |
| 23 | 54,975.635 | 54,984.719 | 9.0 | 0.500 | 1 |

Table S12. ROH islands of the Exmoor Pony

| **Chr.** | **Begin** | **End** | **Length (kb)** | **ROH freq.** | **Known genes** |
| --- | --- | --- | --- | --- | --- |
| 1 | 113,059.953 | 113,939.026 | 879.0 | 0.679 | 2 |
| 1 | 153,630.012 | 153,741.468 | 111.5 | 0.510 | - |
| 2 | 59,469.658 | 60,027.045 | 557.4 | 0.553 | 6 |
| 2 | 97,883.144 | 98,386.038 | 502.9 | 0.535 | - |
| 2 | 99,611.455 | 100,336.718 | 725.3 | 0.615 | - |
| 3 | 65,687.853 | 65,951.617 | 263.8 | 0.510 | - |
| 3 | 105,767.304 | 105,854.172 | 86.9 | 0.510 | 2 |
| 3 | 108,155.338 | 108,674.194 | 518.9 | 0.540 | 1 |
| 4 | 65,432.730 | 66,031.489 | 598.8 | 0.622 | 5 |
| 6 | 50,669.754 | 53,151.551 | 2,481.8 | 0.656 | 20 |
| 6 | 81,197.672 | 81,809.667 | 661.9 | 0.513 | 4 |
| 9 | 44,411.007 | 46,434.207 | 2,023.2 | 0.690 | 10 |
| 11 | 29,229.345 | 30,690.860 | 1,461.5 | 0.592 | 6 |
| 16 | 2,634.563 | 3,230.928 | 596.4 | 0.536 | 5 |
| 16 | 85,718.086 | 85,973.268 | 255.1 | 0.509 | - |
| 18 | 37,632.308 | 37.655.655 | 23.3 | 0.509 | - |
| 18 | 43,405.331 | 44,139.826 | 734.5 | 0.557 | - |
| 18 | 71,126.255 | 72,580.311 | 1,454.0 | 0.563 | 9 |
| 19 | 466.601 | 706.350 | 239.7 | 0.571 | 2 |
| 19 | 3,163.588 | 3,558.119 | 394.5 | 0.513 | 1 |
| 19 | 43,103.485 | 43,485.342 | 381.8 | 0.538 | 1 |
| 22 | 24,562.875 | 25,552.521 | 989.6 | 0.710 | 15 |
| 22 | 26,399.915 | 26,853.848 | 453.9 | 0.513 | 6 |
| 22 | 27,075.347 | 27,269.016 | 193.7 | 0.524 | 6 |
| 22 | 28,985.563 | 30,585.841 | 1,600.2 | 0.559 | 4 |
| 22 | 43,593.112 | 44,276.342 | 683.2 | 0.572 | 8 |
| 22 | 44,335.320 | 44426698 | 91.4 | 0.522 | - |
| 22 | 44,498.568 | 46474498 | 1,975.9 | 0.606 | 17 |
| 22 | 46,498.920 | 46999831 | 500.9 | 0.573 | - |
| 23 | 45,420.463 | 45973756 | 553.3 | 0.610 | 1 |
| 23 | 49,427.817 | 49552039 | 124.2 | 0.503 | 3 |
| 28 | 40,295.281 | 40748701 | 453.4 | 0.525 | 4 |
| 30 | 28,117.484 | 30061306 | 1,943.8 | 0.607 | 32 |
