# Supplementary material for: Genome-Wide Homozygosity Patterns and Evidence for Selection in a Set of European and Near Eastern Horse Breeds
Source: Genes (Basel). 2019 Jun 28;10(7):491. doi: 10.3390/genes10070491 (PMC6679042; doi:10.3390/genes10070491)
Supplement: Supplementary file 1 [file genes-10-00491-s001.zip › Supplementary_file4.doc]

**Supplementary File 4.** Genotype distribution for the *MC1R* locus in the twelve studied breeds (*T/T* = chestnut coat color; *T/C* or *C/C* = non chestnut coat color)

| *MC1R* | *T/T* | *T/C* | *C/C* |
| --- | --- | --- | --- |
| Gidran | 20 | 0 | 0 |
| Haflinger | 78 | 0 | 0 |
| Purebred Arabian | 91 | 53 | 11 |
| Selle Francais | 94 | 143 | 57 |
| French Trotter | 35 | 74 | 46 |
| Posavina | 6 | 9 | 13 |
| Noriker | 36 | 36 | 102 |
| Bosnian Mountain Horse | 2 | 11 | 10 |
| Shagya Arabian | 1 | 9 | 22 |
| Akhal Teke | 7 | 14 | 14 |
| Lipizzan | 4 | 36 | 337 |
| Exmoor Pony | 0 | 1 | 103 |
