# Supplementary material for: Genome-Wide Homozygosity Patterns and Evidence for Selection in a Set of European and Near Eastern Horse Breeds
Source: Genes (Basel). 2019 Jun 28;10(7):491. doi: 10.3390/genes10070491 (PMC6679042; doi:10.3390/genes10070491)
Supplement: Supplementary file 1 [file genes-10-00491-s001.zip › supplementary_file6.doc]

**Supplementary File 6.** Genotype frequencies of SNPs at the loci LCORL/NCAPG, ZFAT and LASP1 for the breeds Bosnian Mountain Horse and Exmoor Pony, both representing the Pony type. (chr. = chromosome; HWE = Hardy-Weinberg equilibrium; n.s. = non significant p<0.05)

| Bosnian Mountain Horse n=23; mean height at withers = 130 - 142 cm; medium to light calibre | | | | | | |
| --- | --- | --- | --- | --- | --- | --- |
| Chr. | SNP |  | position | Genotype Frequency | HWE p-value | Major allele |
| 3 | AX-103666681 | *LCORL/NCAPG* | 105,547.002 | 1/5/17 | n.s. | T (small) |
| 9 | AX-103185455 | *ZFAT* | 75,550.059 | 0/3/20 | n.s. | C (small) |
| 11 | AX-104097126 | *LASP1* | 23,259.732 | 1/9/13 | n.s. | G (small) |
| Exmoor Pony n=104; mean height at withers = 117 – 130 cm; medium calibre | | | | | | |
| Chr. | SNP |  | position | Genotype Frequency | HWE p-value | Major allele |
| 3 | AX-103666681 | *LCORL/NCAPG* | 105,547.002 | 0/0/104 | n.s. | T (small) |
| 9 | AX-103185455 | *ZFAT* | 75,550.059 | 2/6/96 | 0.014 | C (small) |
| 11 | AX-104097126 | *LASP1* | 23,259.732 | 23/53/28 | n.s. | A (big) |
